# RNA sequencing identifies novel regulated IRE1-dependent decay targets that affect multiple myeloma survival and proliferation

**DOI:** 10.1186/s40164-022-00271-4

**Published:** 2022-03-31

**Authors:** Dalia Quwaider, Luis A. Corchete, Marta Martín-Izquierdo, Jesús M. Hernández-Sánchez, Elizabeta A. Rojas, Ignacio J. Cardona-Benavides, Ramón García-Sanz, Ana B. Herrero, Norma C. Gutiérrez

**Affiliations:** 1 Institute of Biomedical Research of Salamanca (IBSAL), Salamanca, Spain; 2 Cancer Research Center-IBMCC (USAL-CSIC), Salamanca, Spain; 3 Hematology Department, University Hospital of Salamanca, Salamanca, Spain; 4 Center for Biomedical Research in Network of Cancer (CIBERONC), Salamanca, Spain; 5 Department of Medicine, University of Salamanca, Salamanca, Spain

**Keywords:** ER stress, UPR, IRE1, RIDD, Multiple myeloma

## Abstract

**Background:**

IRE1 is an unfolded protein response (UPR) sensor with kinase and endonuclease activity. It plays a central role in the endoplasmic reticulum (ER) stress response through unconventional splicing of XBP1 mRNA and regulated IRE1-dependent decay (RIDD). Multiple myeloma (MM) cells are known to exhibit an elevated level of baseline ER stress due to immunoglobulin production, however RIDD activity has not been well studied in this disease. In this study, we aimed to investigate the potential of RNA-sequencing in the identification of novel RIDD targets in MM cells and to analyze the role of these targets in MM cells.

**Methods:**

In vitro IRE1-cleavage assay was combined with RNA sequencing. The expression level of RIDD targets in MM cell lines was measured by real-time RT-PCR and Western blot.

**Results:**

Bioinformatic analysis revealed hundreds of putative IRE1 substrates in the in vitro assay, 32 of which were chosen for further validation. Looking into the secondary structure of IRE1 substrates, we found that the consensus sequences of *IRF4, PRDM1, IKZF1, KLF13, NOTCH1, ATR, DICER, RICTOR, CDK12, FAM168B,* and *CENPF* mRNAs were accompanied by a stem-loop structure essential for IRE1-mediated cleavage. In fact, we show that mRNA and protein levels corresponding to these targets were attenuated in an IRE1-dependent manner by treatment with ER-stress-inducing agents. In addition, a synergistic effect between IMiDs and ER-stress inducers was found.

**Conclusion:**

This study, using RNA sequencing, shows that IRE1 RNase has a broad range of mRNA substrates in myeloma cells and demonstrates for the first time that IRE1 is a key regulator of several proteins of importance in MM survival and proliferation.

**Supplementary Information:**

The online version contains supplementary material available at 10.1186/s40164-022-00271-4.

## Background

The endoplasmic reticulum (ER) plays an essential role in the folding and processing of newly synthesized proteins [1]. Certain environmental stimuli and pathological conditions can produce a quantity of unfolded proteins that exceeds the folding capacity of the ER. Under these circumstances, cells activate the ER stress response or unfolded protein response (UPR), an intracellular signaling pathway that adjusts protein-folding capacity to maintain ER homeostasis [2]. The mammalian UPR is orchestrated by three transmembrane sensors: PERK, ATF6 and IRE1. Activated PERK phosphorylates the eukaryotic translation-initiation factor 2a (eIF2α) to attenuate the rate of general translation initiation and prevent further protein synthesis [3, 4]. Blocking translation during ER stress consequently reduces the protein load in the ER folding machinery [5]. ATF6 migrates into the nucleus and acts as an active transcription factor to upregulate proteins that increase ER folding capacity [6]. Finally, IRE1, a bifunctional enzyme with kinase and endonuclease activity [7], is a master regulator of the UPR pathway. ER stress triggers the oligomerization and trans-autophosphorylation of IRE1, switching on its RNase activity [8, 9]. The primary target of IRE1 is the mRNA that encodes the X-box binding protein (XBP1). In mammalian cells, the full-length mRNA of XBP1, also referred to as the unspliced form (XBP1u), is cleaved by IRE1 at two specific sites, causing the removal of an intron of 26 nucleotides. This splicing event causes a frameshift that allows the translation of the spliced form (XBP1s), which encodes a potent transcription factor [10]. XBP1 induces the expression of various chaperones and other ER-resident proteins, which increases the folding capacity of the ER, and increases the level of membrane phospholipids, triggering its expansion [11]. In addition, activation of IRE1 has also been shown to target several mRNAs for degradation in an XBP1-independent manner. This mechanism, known as regulated IRE1-dependent decay (RIDD), was first demonstrated in *Drosophila melanogaster* [12] and later in yeast species and mammalian cells [13–15]. RIDD is believed to have the potential to selectively relieve the load on the ER by mediating the cleavage and degradation of ER-targeted mRNAs [12]. IRE1 activity has been shown to regulate cell fate decisions, depending on the amplitude and duration of its activation, which, in turn, depends on the intensity of ER stress [16]. RIDD promotes apoptosis by degrading the mRNA-encoding proteins essential for cell survival [16–18] and select microRNAs that normally repress translation of Caspase 2 [18]. It is important to note that the RIDD of miRNAs during ER stress can also modulate the expression of hundreds of mRNA targets [19].

The mechanism by which IRE1 recognizes and cleaves its targets became clear a decade ago, when Oikawa et al. demonstrated that the cleavage site of 13 novel mRNAs RIDD targets contained a consensus sequence (CUGCAG) within a stem-loop structure that was also present in *XBP1* mRNA [20]. Furthermore, it was shown that the consensus cleavage site and stem-loop structure are conserved in mammalian cells, as demonstrated in other known RIDD targets [20–22].

Several studies have suggested that many RIDD targets are yet to be identified, and that these may depend on the nature of the stress stimuli and the tissue context [23]. Multiple myeloma (MM) is a hematological malignancy characterized by the accumulation of clonal plasma cells (PCs) in the bone marrow. The malignant PCs have the ability to produce large amounts of immunoglobulins, which raise the baseline level of ER stress [24] and create a dependency on the UPR for survival [25]. This probably explains the sensitivity of myeloma cells to proteasome inhibitors (PIs) like bortezomib, which exacerbate ER stress [26]. In this regard, MM is a useful model for studying new targets of IRE1. Since BLOC1S1 mRNA has been the only RIDD target identified so far [22], we hypothesized that many other RIDD targets might exist in MM.

In this study, we combined an in vitro cleavage assay with RNA sequencing (RNA-seq) to identify the IRE1-cleavage site of new RIDD targets in MM. We identified hundreds of putative IRE1-mRNA substrates and validated 11 novel stem-loop-containing mRNA targets that were cleaved in vitro and in cells treated with ER stress inducers by IRE1. mRNA and protein levels corresponding to these targets were attenuated under treatment with ER-stress-inducing agents in an IRE1-dependent manner.

## Materials and methods

### Cell lines

The NCI-H929 and MM1S human multiple myeloma cell lines (MMCLs) were acquired from ATCC (American Type Culture Collection). Cell line identity was confirmed periodically by STR analysis with the PowerPlex 16 HS system kit (www.promega.com) and online STR matching analysis (www.dsmz.de/fp/cgi-bin/str.html). Cell lines were cultured in RPMI 1640 medium supplemented with 10% fetal bovine serum and antibiotics (Gibco Life Technologies, Grand Island, NY, USA).

### RNA extraction and in vitro RNA cleavage assay

RNA was extracted from the cell lines using an RNeasy mini kit (Qiagen, Valencia, CA, USA) according to the standard protocol. RNA integrity was assessed using Agilent 2100 Bioanalyzer (Agilent Technologies, Santa Clara, CA, USA) and only RNA preparations with an RNA integrity number (RIN) > 7 were used. For the cleavage assay, total RNA (5 μg) was incubated for 1 h at 37 °C in cleavage buffer (20 mM HEPES, pH 7.5, 50 mM NaCl, 1 mM DTT, and 2 mM ATP) in the presence or absence of 2 μg of recombinant human ERN1/IRE1a (aa 467–977) (Sino Biological Inc., Beijing, China). The reaction was inactivated at 95 °C for 5 min. RNA cleanup of these samples was performed using an RNeasy MinElute Cleanup Kit (Qiagen, Valencia, CA, USA), in accordance with the manufacturer's instructions.

### RNA sequencing

The library for RNA-seq analysis was prepared using approximately 1.0 µg of total RNA from each sample and the TruSeq Stranded mRNA Sample Preparation Kit version 1.0 (Illumina, San Diego, CA, USA), following the manufacturer’s instructions. Briefly, mRNAs were purified using poly-T oligo-attached magnetic beads and the RNA was fragmented. The mRNA fragments were used as templates for first-strand cDNA synthesis by reverse transcription with random hexamers. Upon second-strand cDNA synthesis, double-stranded cDNAs were end-repaired and adenylated at the 3′ ends. Universal adapters were ligated to the cDNA fragments, then the sequencing library of DNA fragments that had adapters on both ends was amplified by PCR, and used to produce the clusters that were then sequenced in an Illumina HiSeq 2000 instrument (Illumina, San Diego, CA, USA). Each sample was sequenced in a separate flow cell lane, producing 26.2–32.4 million paired-end reads, with a final length of 76 bases.

### RNA sequencing analysis

The initial quality control of raw FASTQ files was performed using the FastQC tool (v.0.11.3) (http://www.bioinformatics.babraham.ac.uk/projects/fastqc). Adapter sequences and low-quality bases (Phred score < 20) were trimmed from the raw reads using the Cutadapt (v1.12) program [27]. The processed reads were mapped to the reference human genome GRCh37 (Ensembl annotation, release 87) with the STAR aligner (v.2.5.3a), using default parameters. The resulting BAM alignment files were indexed and sorted using samtools (v.1.3.1) [28].

Raw exon counts were obtained from the BAM files using the dexseq_count Python script available in the DEXSeq package (v. 1.24.4) [29]. Differential exon usage was analyzed analysis using a likelihood ratio (LR) test, and the corresponding graphs produced using the DEXSeq package. Unambiguous exons with a Benjamini & Hochberg false discovery rate (FDR) < 0.05 threshold in the LR test and a negative fold change (FC) < -2 were considered for further analysis. Exons associated with two or more HGNC (HUGO Gene Nomenclature Committee) IDs were considered ambiguous. Pathway overrepresentation was measured with the WebGestalt web tool, using information about the HGNC gene correspondence with the selected exons and the Reactome pathway database [30]. Ensembl mRNA sequences from the transcripts containing the altered exons were used to predict the secondary mRNA structures on the Mfold web server [31]. The predicted secondary structure with the lowest thermodynamic energy was considered the most stable and, therefore, the most biologically likely structure. The dataset is available at the Gene Expression Omnibus (GEO) repository (http://www.ncbi.nlm.nih.gov/geo) under the accession number GSE152070.

### Quantitative reverse transcriptase PCR (qRT-PCR)

Total RNA (500 or 1000 ng) was reverse-transcribed to complementary DNA (cDNA) using SuperScript First-Strand Synthesis System for RT-PCR (Invitrogen, Waltham, MA, USA) with oligo (dt), or using a High-Capacity cDNA Reverse Transcription Kit (Applied Biosystems, Foster City, CA, USA).

Expression of target genes was quantified by qRT-PCR using an iQ SYBR Green Supermix kit (Bio-Rad Laboratories, Hercules, CA, USA) and the primers shown in (Additional file 1: Table S1). PCR melting curves were analyzed to confirm the presence of a single product. Relative gene expression was calculated by the 2^−ΔCt^ method using *GAPDH* as the endogenous control for normalization. Each measurement was performed in triplicate.

### RT-PCR analysis of *XBP1* mRNA splicing

RNA was reverse-transcribed to cDNA using a cDNA Reverse Transcription Kit from Applied Biosystems (Foster City, CA, USA). The PCR reaction was performed using GoTaq (Promega, Madison, WI, USA), following the manufacturer's protocol. The primers used were: *XBP1* forward primer, 5′-TTACGAGAGAAAACTCATGGCC-3′, and *XBP1* reverse primer, 5′-GGGTCCAAGTTGTCCAGAATGC-3′. The cycling conditions were 95 °C for 5 min, followed by 35 cycles of 95 °C for 1 min, 58 °C for 30 s, and 72 °C for 30 s, then 72 °C for 5 min. Electrophoresis was performed using 5 μl per sample in a 2% agarose gel.

### ER stress induction

To induce ER stress, cells were seeded at a density of 1 × 10^6^ cells/ml (NCI-H929 and MM1S) and incubated for 4 or 16 h with the following inducers: thapsigargin (1.5 µM), tunicamycin (10 µg/ml), brefeldin A (600 ng/ml) (Sigma-Aldrich, St Louis, MO, USA), and dithiothreitol (2 µM) (Promega, Madison, WI, USA). To inhibit IRE1 RNase activity, cells were treated with 15 μM 4μ8c (MedChemExpress, Monmouth Junction, NJ, USA).

### Western blot

Whole cell lysates were collected using RIPA buffer (Sigma-Aldrich, St Louis, MO, USA) containing protease inhibitors (Complete Protease Inhibitor Cocktail Tablets; Santa Cruz Biotechnology, Delaware, CA, USA) and phosphatase inhibitors (Phosphatase Inhibitor Cocktail A and B; Santa Cruz Biotechnology, Delaware, CA, USA). Protein concentration was measured using the Bradford assay (BioRad Laboratories, Hercules, CA, USA). Protein samples (30 μg/lane) were subjected to sodium dodecyl sulfate–polyacrylamide gel electrophoresis (SDS-PAGE) and transferred to 0.45-nm polyvinylidene fluoride (PVDF) membranes (BioRad Laboratories, Hercules, CA, USA). The primary antibodies used for immunoblotting were: anti-IRF4 and anti-DICER from Santa Cruz Biotechnology (Delaware, CA, USA); anti-KLF13 from Novus Biologicals (Centennial, CO, USA); anti-ATR and anti-CENPF from GeneTex (Irvine, CA, USA); and anti-Blimp1, anti-Ikaros, anti-NOTCH1 and anti-GAPDH (used as an internal control for protein loading) from Cell Signaling Technology (Beverly, MA, USA). The membranes were then washed and incubated with the corresponding secondary horseradish peroxidase-linked antibodies: anti-mouse IgG, anti-rabbit IgG antibodies (Abcam, Cambridge, UK) or anti-goat IgG (Santa Cruz Biotechnology, Delaware, CA, USA) at 1:10,000. Chemiluminescence was detected using the Amersham ECL Plus WesternBlotting Detection Reagent (GE Healthcare, Chicago, IL, USA).

### Cell transfection

MM cell line transfections were carried out as previously detailed [32]. Cells were transfected with on-TARGET plus control pool or on-TARGET plus SMART pool Human of XBP1 (Dharmacon, Lafayette, CO, USA).

### Cell viability assays

MMCLs were seeded into 96-well plates (30.000 cells/well) and were treated with different concentrations of IMiDs; pomalidomide or lenalidomide (0.1, 1, and 10 μM) or ER inducers; tunicamycin (100, 200, and 500 nM), thapsigargin in H929 (1.5, 2, and 2.5 nM) or in MM1S (1, 1.5, and 2 nM) for 48 h. Cell proliferation was determined using 3-(4,5-dimethylthiazol-2-yl)- 2,5-diphenyl-2H-tetrazolium bromide (MTT) (Sigma-Aldrich). MTT was dissolved in PBS (5 mg/mL) and 10 μL of this salt per well was added to cells. After 1 h of incubation formazan crystals were dissolved in DMSO (100 μL/well). Absorbance was measured at 570 nm in a plate reader (Ultra Evolution, Tecan). Four wells were analyzed for each condition. The results are presented as the mean ± SD of quadruplicates of a representative experiment that was repeated at least twice.

## Results

### Identification of RIDD targets in MM by RNA sequencing

**Fig. 1:**
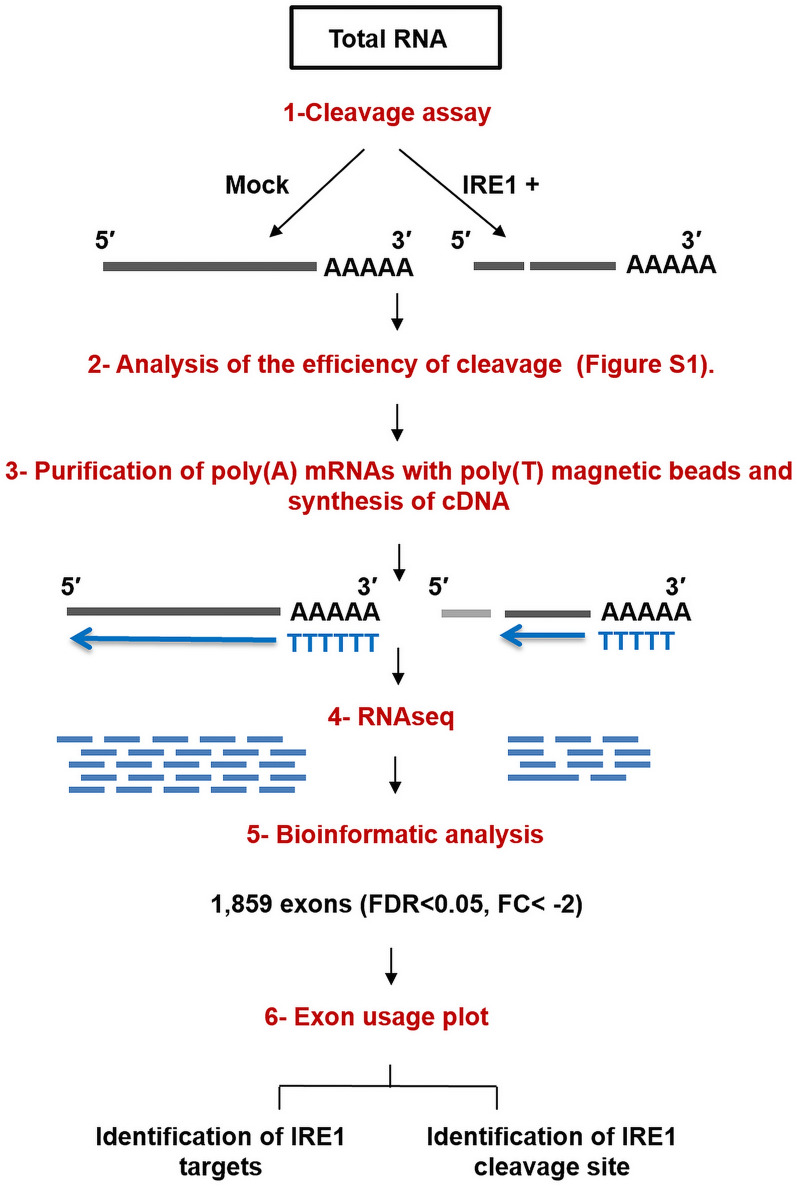
Schematic representation of the cleavage reaction and RNA-sequencing procedure. Total RNA from H929 cells was subjected to in vitro cleavage by the cytoplasmic domain of human IRE1 or to a mock treatment. The RNA was reverse-transcribed with oligo-dT primers and then RNA-seq was performed. The lost 5´ side of the IRE1-mRNA substrates, represented in gray, is not reverse-transcribed, producing fewer reads in the mRNA cleaved by IRE1 than in mock treatment samples

To identify mRNAs cleaved by IRE1 in MM we followed the steps shown in Fig. 1. An in vitro cleavage assay of the total RNA obtained from the H929 cell line was carried out in the presence or absence of recombinant IRE1 protein. To analyze the efficiency of the cleavage reaction, a part of mRNAs present in three IRE1-treated samples and three mock-treated controls were reverse-transcribed using an oligo(dT) primer, and mRNA levels of two known IRE1 targets, XBP1 and BLOC1S1 [22], were quantified by qRT-PCR (Additional file 2: Fig. S1). Using a pair of primers that annealed on both sides of the IRE1-cleaving sites in the tested molecules, markedly lower XBP1 and BLOC1S1 mRNA levels were observed in the three samples treated with IRE1, as expected. Then, we carried out RNA-sequencing from purified poly (A)-containing mRNAs. In this manner, reads mapping entire mRNA molecules were obtained from control samples. Conversely, IRE1-mRNA substrates lost the 5´ mRNA fragments during the purification process, which reduced the number of reads in these regions (Fig. 1) giving rise to a scarcity of several exons. A bioinformatic analysis identified 1,859 unambiguous exons (Additional file 3: Table S2), corresponding to 863 HGNC-annotated genes. These exhibited significantly lower read counts in IRE1-treated samples than in controls (FDR < 0.05, FC < -2). XBP1 was ranked 14^th^ among the underexpressed exons, based on FC (Additional file 3: Table S2, K column). Reactome pathway enrichment analysis revealed that the putative mRNA targets were involved in a variety of pathways, such as membrane trafficking, cell cycle, DNA repair, and ubiquitination and proteasome degradation (Additional file 4: Fig. S2).

**Fig. 2:**
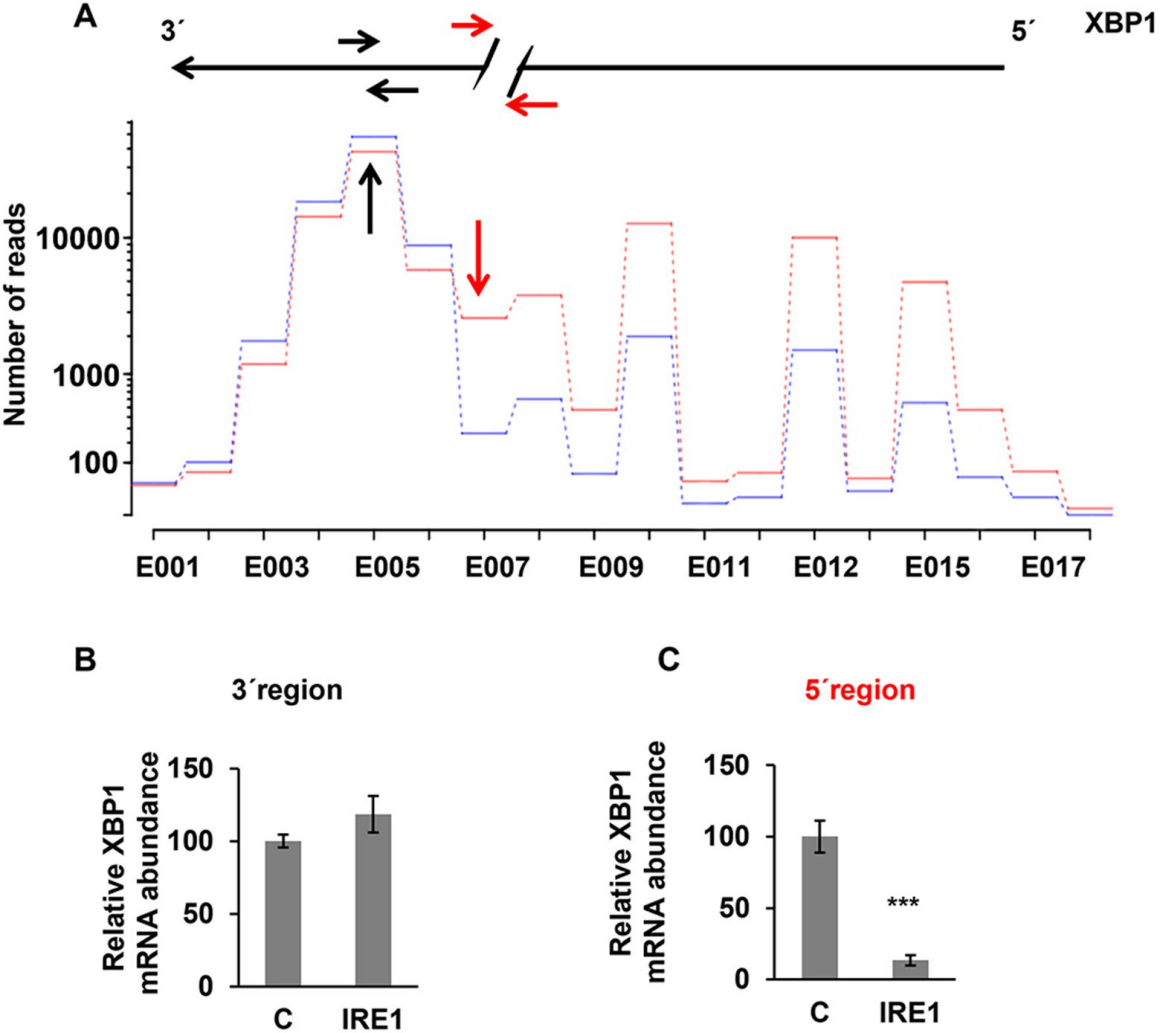
Differential exon usage of *XBP1* according to RNA-seq data. **A** Exon-usage plots of *XBP1* mRNA, which represents the number of reads in mock (red) and IRE1-treated (blue) samples. **B** XBP1 mRNA measured by qRT-PCR using the cDNAs synthesized with oligo (dT) and primers mapping the 3´ region (black arrow). **C** XBP1 mRNA measured by qRT-PCR using the cDNAs synthesized with oligo (dT) and primers mapping the 5´ region (red arrow). All results are presented as the means ± SD of three experiments. (***p < 0.001)

We plotted the exon usage values of the 863 genes, which allowed us to visualize all the exons in the 5´ region of the mRNAs with fewer reads in IRE1-treated samples than in controls. The exon-usage plot corresponding to the XBP1 mRNA showed that the number of reads corresponding to exons located 5′ to the cleavage sites was clearly lower in IRE1-treated compared with untreated controls, whereas the number of reads was similar for the exons located in the 3´ region of both samples (Fig. 2A). These findings were then validated by qRT-PCR using the cDNAs synthetized with oligo (dT) and a pair of primers that map the 3´ region of the XBP1 mRNA molecule (Fig. 2B, black arrows), or on both sides of the IRE1-cleavage sites on 5´region (Fig. 2C, red arrows).

**Fig. 3:**
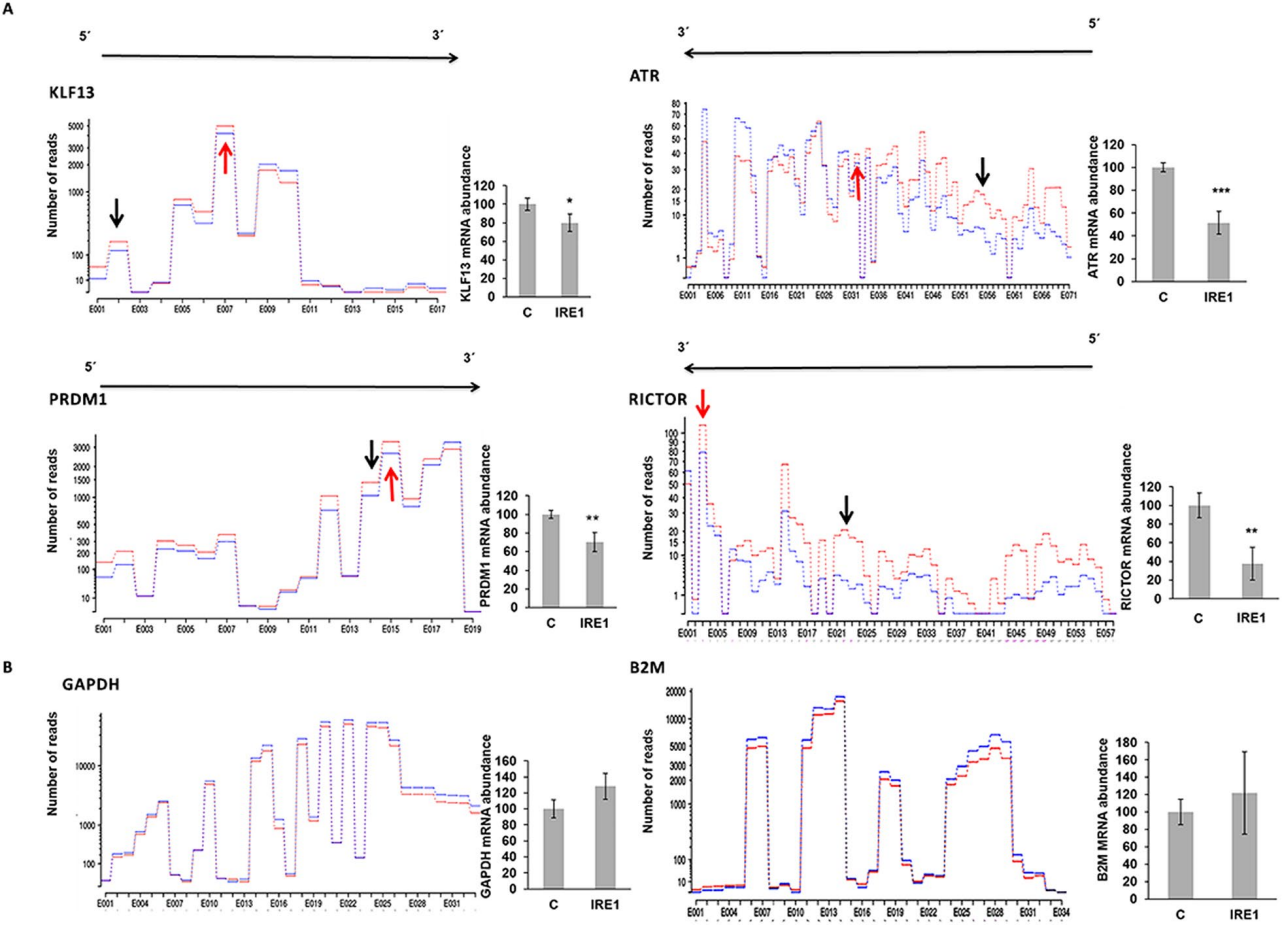
Validation of putative IRE1 substrates. **A** Exon-usage plots of *KLF13, ATR, PRDM1,* and *RICTOR* mRNAs, showing the number of reads in mock (red) and IRE1-treated (blue) samples. The black arrows represent the site of primers used in the 5´ region of the putative IRE1 substrates. Red arrows indicate the site of primers mapping the predicted cleavage site. Right panel of each exon-usage plot shows the abundance of *KLF13, ATR, PRDM1*, and *RICTOR* mRNA measured by qRT-PCR using the cDNAs synthesized with oligo (dT). **B** *GAPDH* and *B2M* were used as negative controls, and their mRNA was measured by qRT-PCR. All results are presented as the means ± SD of three experiments. (*p < 0.05, **p < 0.01, ***p < 0.001)

Of the 863 genes with an FC < -2, we selected for validation those exhibiting the greatest FC between the control samples and those treated with IRE1, and in turn those that showed a similar exon-usage plot to that of XBP1. A total of 30 genes were selected by these criteria (Table 1). We also included *IRF4* and *PRDM1* genes in the validation analysis, based on their known function in PC differentiation and in the pathogenesis of MM, even though both genes had a FC > -2 (Table 1). We designed primers complementary to the 5´ region of the putative IRE1 substrates (Fig. 3A, marked with a black arrow) and measured the amount of mRNA after the in vitro cleavage reaction by qRT-PCR. We found that the 5´ regions of the mRNAs from all 32 IRE1-substrate candidates significantly decreased in IRE1-treated samples compared to controls (four representative examples are shown in Fig. 3A; the others are illustrated in Additional file 5: Fig. S3). Notably, IRE1 did not cleave the mRNA of the housekeeping genes *GAPDH* and *B2M*, demonstrating that this protein is not a random nonspecific RNase (Fig. 3B).

**Table 1:** Selected putative IRE1 substrates

Gene symbol	FC	Function
ATM	− 34.9	Regulation of DNA damage response
VPS13C	− 15.96	Regulation of mitocondrial function
BIRC6	− 12.04	Ubiquitination and proteosomal protein involved in ubiquitin activity
KMT2C	− 9.94	Protein methylation
HUWE1	− 7.91	Ubiquitination and proteosomal protein involved in ubiquitin activity
RICTOR	− 6.79	TOR signaling
CDK12	− 6.55	Cyclin dependent kinase 12
CENPF	− 6.03	Cell cycle regulation and apoptosis induction
VPS13D	− 6.01	Membrane trafficking
GOLGB1	− 6.00	Golgi to plasma membrane transport
FAM168B	− 5.81	Inhibitor
ATR	− 5.60	Regualtion of DNA damage response
AKAP9	− 5.34	Cell cycle regulation and apoptosis induction
NOTCH1	− 4.86	Transcription factors (Cell fate determination, proliferation, differentiation, apoptosis)
PCM1	− 3.84	Cell cycle regulation and apoptosis induction
UBR2	− 3.60	Ubiquitination and proteosomal protein involved in ubiquitin activity
KLF13	− 3.44	Transcription factors (Cell fate determination, proliferation, differentiation, apoptosis)
mTOR	− 3.03	TOR signaling
DICER	− 2.99	Ribonuclease III activity
XRN1	− 2.90	Regualtion of DNA damage response
CUL9	− 2.81	Ubiquitination and proteosomal protein involved in ubiquitin activity
UBE4B	− 2.68	Ubiquitination and proteosomal protein involved in ubiquitin activity
PSME4	− 2.47	Ubiquitination and proteosomal protein involved in ubiquitin activity
CUL4B	− 2.36	Ubiquitination and proteosomal protein involved in ubiquitin activity
UBR3	− 2.35	Ubiquitination and proteosomal protein involved in ubiquitin activity
UBA6	− 2.33	Ubiquitination and proteosomal protein involved in ubiquitin activity
IKZF1	− 2.28	Transcription factors (Cell fate determination, proliferation, differentiation, apoptosis)
PSMD1	− 2.24	Ubiquitination and proteosomal protein involved in ubiquitin activity
CUL5	− 2.05	Ubiquitination and proteosomal protein involved in ubiquitin activity
ERAP1	− 2.00	Protein processing and transport. Cleaves other proteins into smaller fragments
PRDM1	− 1.71	Transcription factor implicated in the differentiation of PC
IRF4	− 1.59	Transcription factor implicated in the differentiation of PC

Together, these results indicate that RNA-seq is a suitable method for identifying new targets of IRE1 in the cells.

### Identification of the IRE1 cleavage site in RIDD targets

**Fig. 4:**
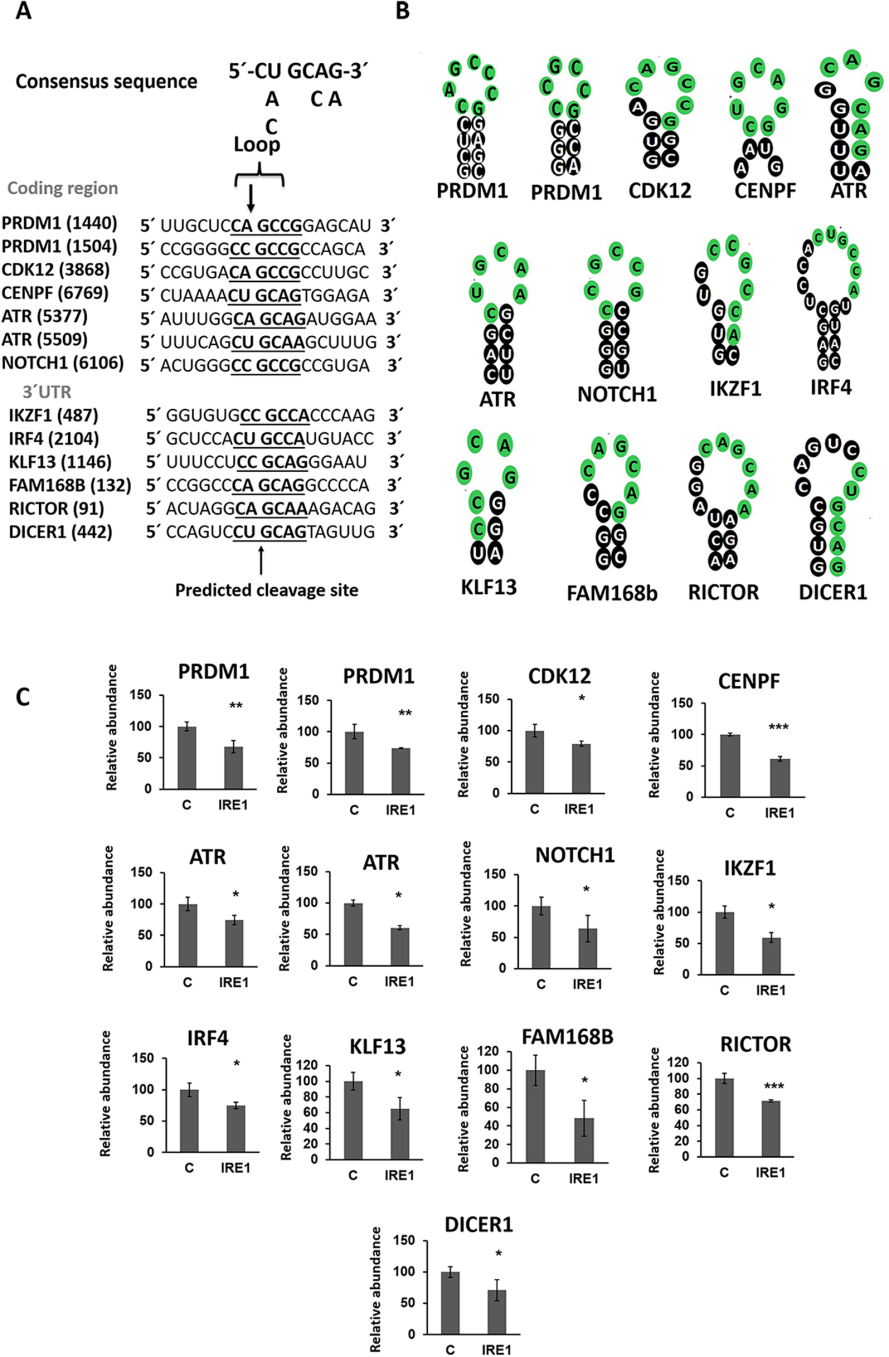
Mapping the cleavage site of IRE1 targets. **A** mRNA sequence around the consensus sequence of the newly identified IRE1 targets. The consensus sequence is underlined. The numbers indicate the guanine position at the consensus sequence, where IRE1 is supposed to cleave. **B** Schematic representation of the secondary mRNA structures containing the consensus sequence of the identified targets of IRE1. **C** mRNA levels of the indicated genes in H929 determined by qRT-PCR. cDNAs were obtained from mRNAs cleaved by IRE1 or mock treatment samples (control, **C**) and synthesized with oligo (dT) and primers mapping the predicted cleavage site. All results are presented as the means ± SD of three experiments. (*p < 0.05, **p < 0.01, ***p < 0.001)

It has been shown that the cleavage activity of IRE1 is a sequence-specific event occurring at the XBP1-like consensus site [20, 22]. We examined whether the newly identified IRE1 targets contained the consensus CUGCAG sequence within an XBP1-like stem loop structure [20]. First, the secondary structures of the 32 mRNAs identified as IRE1 substrate candidates were obtained using Mfold, and then a search for stem-loop structures near the cleavage regions was undertaken. The cleavage region for each IRE1-target mRNA was identified from the previously generated exon-usage plots, since it must correspond to the first region that exhibits fewer reads in IRE1-treated samples than in mock samples (Fig. 3 and Additional file 5: Fig. S3, red arrows). Some molecules displayed putative loops, but outside the identified IRE1-cleavage site. Following this procedure, we found that 11 of the 32 mRNAs (34%) exhibited a clear stem-loop structure that contained the consensus sequence around the IRE1-cleavage site (Fig. 4A, B). Some of the targets exhibited the IRE1-cleavage site in the coding regions, whereas others have the XBP1-like stem loop at the 3´ UTR region. On the other hand, most of the mRNAs contained one cleavage site; *PRDM1* and *ATR* contained two putative cleavage sites located close to each other (Fig. 4A, B).

To confirm that the 11 mRNAs were cleaved by IRE1 at the consensus sequence within the loop structure, we designed primers on both sides of this sequence and performed qRT-PCR (Fig. 3A and Additional file 5: Fig. S3, red arrows). We found that the loop region of these mRNAs was lost in these targets according to the PCR results, which revealed that the mRNA levels of all 11 IRE1 targets were significantly lower in IRE1-treated samples than in the controls (Fig. 4C)**.**

### IRE1 degrades the identified RIDD targets in MM in response to ER stress

**Fig. 5:**
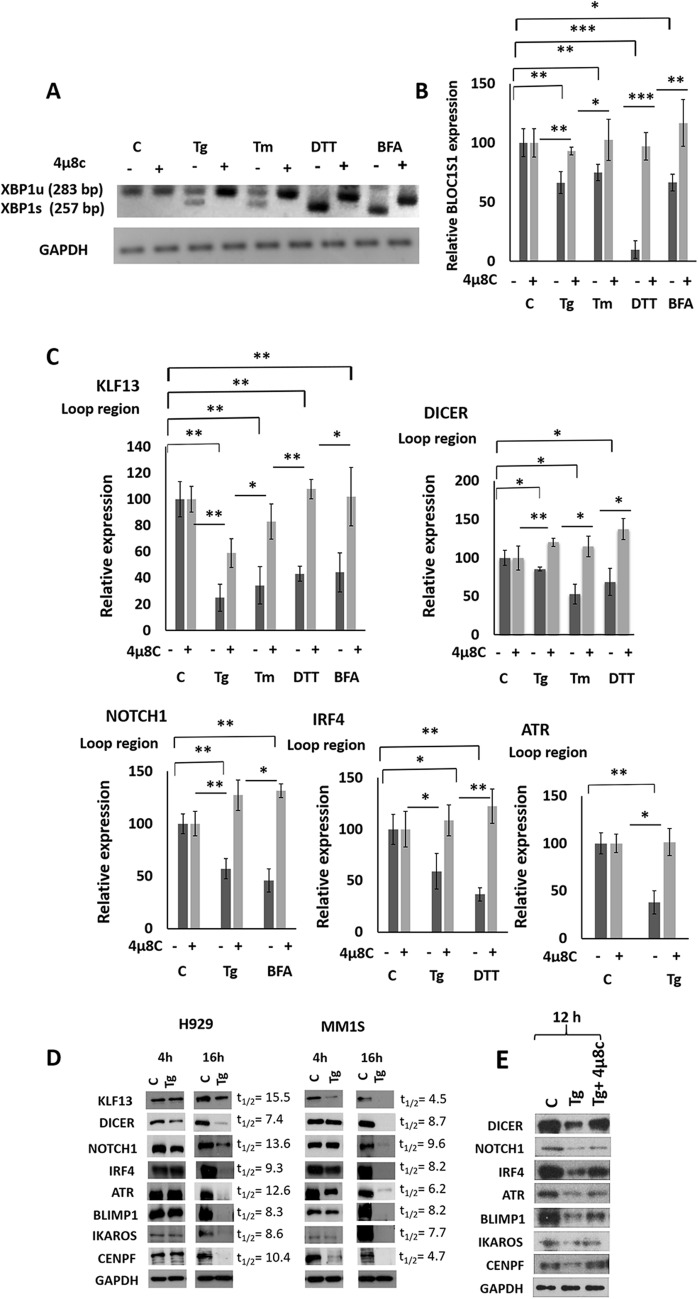
RIDD targets in MM. **A** Representative agarose gel electrophoresis of the PCR product surrounding the XBP1 splice site in H929. **B** *BLOC1S1* mRNA measured by qRT-PCR. **C** mRNA levels of the indicated genes were determined by qRT-PCR. H929 cells were treated with different ER-stress-inducers in the presence or absence of 4μ8c. **D** Levels of IRE1 substrates in H929 and MM1S after 4 h and 16 h of thapsigargin treatment. The amount of each protein was quantified by densitometry, normalized to GAPDH and plotted against time to determine the half-lives of IRE1 substrate proteins (t1/2). **E** Protein levels of the indicated genes in H929 12 h after the treatment with thapsigargin in the presence or absence of the 4μ8c. All results are presented as the means ± SD of three experiments. (*p < 0.05, **p < 0.01, ***p < 0.001)

There is controversy over whether the targets of IRE1 cleavage are degraded upon ER stress induction. This has been observed for *BLOC1S1* and *CD59* mRNA targets [21, 22], but not for others [20]. We investigated this aspect in the 11 RIDD targets identified in this study by treating H929 cells with one of four ER stress inducers (thapsigargin, tunicamycin, dithiothreitol or brefeldin A) for 4 h [33]. To confirm that the putative mRNA degradation was IRE1-dependent, the experiment was carried out in the presence or absence of the IRE1 RNase inhibitor 4µ8c [34]. Previously, the induction of ER stress in H929 cells had been verified by the analysis of the spliced/unspliced forms of *XBP1* (Fig. 5A), and by measuring the levels of *BLOC1S1* mRNA (Fig. 5B). All four agents clearly induced the splicing of XBP1 and reduced BLOC1S1 mRNA levels in an IRE1-dependent manner, since both effects were reverted in the presence of 4µ8c.

We quantified the 11 targets by qRT-PCR using the primers surrounding the previously identified cleavage sites. We found that the mRNA levels of KLF13, DICER, NOTCH1, IRF4, and ATR were reduced upon treatment with some of the ER inducers, these effects were not observed in the presence of 4µ8c (Fig. 5C). No stress-dependent decay was detected with the other RIDD targets under the conditions assayed. To demonstrate that the identified targets were not transcriptionally downregulated by XBP1 we silenced XBP1 expression using small interfering RNA (siRNA) (Additional file 6: Fig. S4 A and B). Then we performed qRT-PCR using RNA obtained from cells treated or not with thapsigargin. As shown in the Additional file 6: Figure S4 C, treatment with this ER stress inducer decreased the abundance of the identified IRE1 targets, not only in cells transfected with the siRNA non-targeting control, but also in those transfected with the XBP1 siRNA. These results indicate that the downregulation of the identified target mRNAs is ER-stress dependent but XBP1 independent.

We also analyzed the effect of ER-stress induced by thapsigargin at the protein level using western blot at different times. We found that KLF13, DICER, NOTCH1, IRF4, and ATR protein expression had not changed by 4 h post-treatment, whereas it had clearly decreased by 16 h (Fig. 5D). This result prompted us to speculate that the effect of stress-dependent decay could be better detected after a longer time. Consequently, we analyzed protein levels of BLIMP1 (encoded by *PRDM1*), IKAROS (encoded by *IZKF1*) and CENPF, whose mRNAs did not display decay 4 h post treatment, after a long exposure (16 h) to thapsigargin. As shown in Fig. 5D, the levels of these proteins were also lower in thapsigargin-treated cells than in the untreated controls. The FAM168B and CDK12 proteins could not be analyzed because the commercial antibodies were no sufficiently specific.

Finally, we decided to check the protein levels of the newly identified RIDD targets in another MM cell line with a different genetic background, like MM1S, after thapsigargin exposure for 4 and 16 h. Similar to the results obtained in H929, protein levels of RIDD substrates had not changed after 4 h, with the exception of KLF13 and CENPF, but had clearly decreased after 16 h of thapsigargin treatment (Fig. 5D). Importantly, levels of GAPDH did not change during the course of the experiment, again revealing that GAPDH mRNA is not an IRE1 target, and that IRE1 is not a random non-specific RNase.

To confirm that the reduction of protein levels of the identified RIDD targets was due to IRE1 RNase activity and not only to a translational attenuation coupled to the chemical induction of ER stress, H929 cells were treated with thapsigargin in the presence or absence of the IRE1 RNase inhibitor and new western blots were carried out. As shown in the Fig. 5E the levels of the indicated proteins were lower in thapsigargin-treated cells than in the untreated controls, while this effect was partially reverted by the treatment with the IRE1 inhibitor. These results confirm that the stress-induced degradation of these targets is due to IRE1-RNase activity.

Together, our results show that the mRNAs of *KLF13, DICER, NOTCH1, IRF4, ATR, BLIMP1, IKAROS,* and *CENPF* genes are novel RIDD targets in MM and that the stress-induced degradation of these mRNAs is IRE1-dependent.

### IMiDs in combination with ER-stress inducers results in synergistic anti-myeloma effect

The fact that two of the identified RIDD targets, IRF4 and IKZF1, are targeted by IMiDs, made us hypothesize that the combination of these drugs with ER-stress inducers (that also lowered IRF4 and IKZF1 levels) could exert a synergistic anti-myeloma effect. To explore this potential synergy, MM cell lines were treated for 48 h with different doses of pomalidomide or lenalidomide with different concentrations of thapsigargin or tunicamycin, and viability was analyzed by MTT assay (Additional file 7: Fig. S5). The combination indices (CIs), calculated with the Compusyn software, were below 1 in H929 and MM1S cell lines, revealing a synergistic interaction between IMiDs and ER-stress inducers (Additional file 7: Fig. S5). The synergism of the double combination was stronger in H929 than in MM1S, since lower CIs at all doses were obtained (Additional file 7: Fig. S5A and B).

## Discussion

IRE1 is a key component of cell fate switch because it produces either adaptive/pro-survival or death signals, depending on the intensity and duration of ER stress. This occurs through unconventional splicing of XBP1 mRNA, and by RIDD [35, 36]. In this study, we have identified novel RIDD targets and their IRE1-cleavage sites in MM cell lines through a combination of in vitro cleavage assay and RNA-sequencing. We found that *IRF4, PRDM1, IKZF1, KLF13, NOTCH1, ATR, DICER, RICTOR, CDK12, FAM168B,* and *CENPF* mRNAs had the consensus sequence (CUGCAG) accompanied by a stem-loop structure essential for IRE1-mediated cleavage. In addition, we show that all these targets were degraded in MM cells after induction of ER stress by IRE1. The cleavage activity of IRE1 is a sequence-specific event occurring within the XBP1-like stem structure [20]. The consensus sequence and the stem loop structure are also conserved in previously identified IRE1 targets, such as *CD59*, *Sparc*, and *PER1,* which also showed mRNA decay in an IRE1-dependent manner [21, 37, 38]. In myeloma cells, the only RIDD target that had already been identified was BLOC1S1 mRNA, which contains an XBP1-like stem loop, and is degraded upon ER stress induction by IRE1 at guanine 444 [22]. While we also confirmed this finding, the novel contribution of our study is to identify several key genes in MM as RIDD targets by taking advantage of the greater potential and sensitivity of RNA-seq. In addition, the IRE1-dependent degradation of these targets was demonstrated in MM cells exposed to ER stress. Nevertheless, it is important to point out that the in-vitro cleavage assay used in our experiments also has some limitations, such as the use of a truncated IRE1, the absence of any spatial–temporal organization of IRE1 and mRNAs, and the inability to detect RIDD targets lacking poly(A) tails.

RIDD activity becomes cytotoxic after prolonged and unmitigated exposure to ER stress [36]. Some of the RIDD targets identified in this study, such as *NOTCH1*, *DICER, IRF4*, and *IKZF1*, encode proteins involved in the survival/proliferation of MM cells [39–43], so their ER stress-mediated degradation may promote cell death. For example, NOTCH1 inhibition in MM induces apoptosis and reduces the proliferation rate, and the knockdown of DICER significantly decreased growth and viability of myeloma cells [39–41]. NOTCH blockade also makes MM cells more sensitive to standard chemotherapies [39] and to pro-apoptotic compounds such as Bcl-2/Bcl-XL inhibitors [44]. It had been previously shown that ER stress reduces DNA double-strand break (DSB) repair and increases radiosensitivity of tumor cells via proteasomal degradation of Rad51 [45]. Here, we found that *ATR*, which has a well-established role in the signaling and repair of DSBs [46] is a target of IRE1. We found that the mRNA encoding other proteins with essential roles in DNA damage signaling and repair, such as ATM, Rad50, and Xrn1 (Additional file 3: Table S2), were also downregulated upon treatment with the IRE1 recombinant enzyme. These results suggest that ER stress might prevent DSB repair by several means, which might act as part of a pro-apoptotic signaling mechanism that is triggered by severe DNA damage.

Some other RIDD targets validated in this study, such as *PRDM1*, *IRF4* and *IKZF1*, are mRNAs involved in B cell maturation. BLIMP1 and IRF4 along with XBP1 are the three pillars that maintain plasma cell differentiation status [47], and IKAROS is a transcription factor that regulates early-lymphoid cell development and promotes B-cell lineage maturation [48]. Expression of BLIMP1 or XBP1 is sufficient to drive BC differentiation towards Ig-secreting PCs, although their expression is not required for PC survival [49, 50]. In contrast, the survival of PC critically depends on IRF4 [42], and IKAROS knockdown inhibits proliferation and induces apoptosis of MM cell lines [43]. In fact, IKAROS is one of the proteins most strongly downregulated after treatment with immunomodulatory drugs (IMiDs) and it must be degraded for these agents to exert their cytotoxic effect [51–53]. Some studies have revealed that patients with a low level of *IKZF1* expression had significantly better survival than those with a higher level of expression. It has also been suggested that MM cells with a low level of IKAROS expression are more sensitive to therapy [54, 55]. The analysis of the mechanisms regulating IKAROS expression may be particularly important for understanding how resistance to IMiDs develops [53]. For our part, we found that IKAROS downregulation may be the result of the induction of ER stress. Interestingly, we found a synergistic interaction between the IMiDs and the ER-stress inducers, which may provide avenues for further research.

The relevance of XBP1s to MM pathogenesis has been previously reported [50]. Two other studies have demonstrated that the blockade of IRE1-XBP1 axis by IRE1 alpha inhibitors induces anti-MM cytotoxicity and enhances sensitivity of MM cells to PI [56, 57]. Conversely, another study showed that silencing of either IRE1 or XBP1 not only failed to impair MM cell growth, but also promoted resistance to bortezomib [50]. The authors found that the weaker response to bortezomib was related to maturation arrest in the plasma cells, as indicated by the repression of plasma cell maturation markers, the smaller quantity of immunoglobulin produced, and the lower levels of UPR activation. In keeping with this, a low level of *XBP1* gene expression in MM patients has been associated with poor response to bortezomib treatment [58]. Our results are consistent with this line of research in the sense that the RNase activity of IRE1 induced degradation of proteins involved in MM cell proliferation. Therefore, the role of IRE1 in maintaining plasma cell differentiation and secretory immunoglobulin production in addition to the downregulation of proteins and transcription factors that sustain MM cell survival, suggest that IRE1 inhibitors may not be a proper approach for treating MM. Further research to test the potential of IRE1 activity as a predictive biomarker of sensitivity to anti-myeloma therapy is needed.

## Conclusion

This study, using RNA sequencing, shows that IRE1 RNase has a broad range of mRNA substrates in myeloma cells. The results demonstrate for the first time that IRE1 regulates several proteins of key significance in multiple myeloma survival and proliferation. These data support further research aimed to test the potential of IRE1 activity as a predictive biomarker of sensitivity to anti-myeloma therapy.

## Supplementary Information

**Additional file 1: Table S1.** List of primer sequences used for RT-PCR analysis. F: Forward primer. R: Reverse primer.

**Additional file 2: Fig. S1. **Efficiency of cleavage reaction. (A) XBP1 and (B) BLOC1S1 mRNA levels measured by qRT-PCR using the cDNAs synthesized with oligo (dT), and primers mapping the cleavage site of IRE1 from mock and IRE1-treated samples.

**Additional file 3: Table S2.** List of differential usage of exons from RNA-seq.

**Additional file 4: Fig. S2. **Reactome pathway analysis using RNA-seq data. Bar chart representing the most significantly enriched pathways. FDR ≤ 0.05.

**Additional file 5: Fig. S3. **Validation of putative IRE1 substrates. Exon-usage plots of the 28 remaining putative mRNAs, showing the number of reads in mock (red) and IRE1-treated (blue) samples. The black arrows represent the site of primers used in the 5´ region of the putative IRE1-substrates. Red arrows represent the site of primers mapping the predicted cleavage site. Right panel of each exon-usage plot shows the abundance of mRNA in the corresponding target. All results are presented as the means ± SD of three experiments. (*p < 0.05, **p < 0.01, ***p < 0.001).

**Additional file 6: Fig. S4. **XBP1 knockdown. (A) mRNA levels of XBP1 in H929 determined by qRT-PCR 48 h after transfection with XBP1 siRNA. (B) Western blot of XBP1 in H929. (C) mRNA levels of the indicated genes in XBP1 knockdown cells determined by qRT-PCR. H929 cells were treated in the presence or absence of thapsigargin. All results are presented as the means ± SD of three experiments. (*p < 0.05, **p < 0.01, ***p < 0.001). “NS” indicates not significant (p > 0.05).

**Additional file 7: Fig. S5. **Synergistic effect of ER-stress inducers and IMiDs treatment in MMCLs. (A) H929 and (B) MM1S cells were exposed for 48 h to the indicated concentrations of ER- stress inducers and IMiDs, and cell viability assay was assessed by MTT. CI values less than 1 indicated a synergistic effect. These values were calculated using Compusyn Software. C: control (untreated cells). IMiDs; poma (pomalidomide) or lena (lenalidomide). ER inducers; Tm (tunicamycin) or Tg (thapsigargin).

## Data Availability

All data generated or analyzed during this study are included in this manuscript.

## Abbreviations

ER: Endoplasmic reticulum
UPR: Unfolded protein response
XBP1: X-box binding protein
XBP1u: XBP1 unspliced form
XBP1s: XBP1 spliced form
RIDD: Regulated IRE1-dependent decay
MM: Multiple myeloma
PC: Plasma cells
RNA-seq: RNA sequencing
DSB: DNA double-strand break
