# Supplementary material for: RNA sequencing identifies novel regulated IRE1-dependent decay targets that affect multiple myeloma survival and proliferation
Source: Exp Hematol Oncol. 2022 Mar 31;11:18. doi: 10.1186/s40164-022-00271-4 (PMC8969279; doi:10.1186/s40164-022-00271-4)
Supplement: Supplementary file 1 — Additional file 1: Table S1. List of primer sequences used for RT-PCR analysis. F: Forward primer. R: Reverse primer. [file 40164_2022_271_MOESM1_ESM.docx]

**Supplementary Table 1**. List of primer sequences used for RT-PCR analysis in this study.

F: Forward primer. R: Reverse primer

|  | **Primer name** | **Primer sequence (5´-3´)** |
| --- | --- | --- |
| **VPS13C** | **VPS13C F** | TGGACAGACTCAAAGCTCTCT |
|  | **VPS13C R** | CCTGGTTGATATCATTGTTATAAA |
| **ATM** | **ATM F** | GCAGAAACACTCCCAGCTT |
|  | **ATM R** | AGGTGTTTGGTGAGAATGTCC |
| **BIRC6** | **BIRC6 F** | TCAAAGAGGGAACCTTCCAA |
|  | **BIRC6 R** | GGACATGGAGACTGAAGAAACA |
| **HUWE1** | **HUWE1 F** | TGATGAAGGTGAAGAGGGAGA |
|  | **HUWE1 R** | CATGTTGTCAAACTCAATGATGA |
| **CDK12** | **CDK12 F** | CAGTTCACGCAGTCGTCATT |
|  | **CDK12 R** | GGGGCAGATTTTTCCAATTT |
|  | **CDK12 LOOP F** | TCTGGTTGAAGGCGATCTTT |
|  | **CDK12 LOOP R** | GTTTCAGGCCCATCAGTGTT |
| **VPS13D** | **VPS13D F** | AGTGGGCATGGCAAATAGAG |
|  | **VPS13D R** | CAGAATTCCCAATGCTGACA |
| **GOLGB1** | **GOLGB1 F** | TGAGGAGAATATTGCTTCTTTGC |
|  | **GOLGB1 R** | CTCAGCTTTCAGCTCCTCCA |
| **FAM168B** | **FAM168B F** | CTGTATGCAGCACCTCCTCA |
|  | **FAM168B R** | GTGACCCCGTTGCCTCTA |
|  | **FAM168B LOOP F** | ATTATTGGGGATTCCACAGC |
|  | **FAM168B LOOP R** | CAGGCAGTCCACAAAACAGA |
| **NOTCH1** | **NOTCH1 F** | AGAATGACGCTCGTACCTGC |
|  | **NOTCH1 R** | ACAAGAGCCCGTTGAATTTG |
|  | **NOTCH1 LOOP F** | GCTGGAGGACCTCATCAACT |
|  | **NOTCH1 LOOP R** | TCTCCTCCCTGTTGTTCTGC |
| **UBR2** | **UBR2 F** | GGATCCTCTTGTTCATTTATCAGAA |
|  | **UBR2 R** | TCTGCTGGCAATTCACTTTC |
| **mTOR** | **mTOR F** | AGTCAAGAGGAGTCTACTCGC |
|  | **mTOR R** | GCCAAGATGCCACCTTTCCT |
| **DICER** | **DICER F** | TGGCAAACAAGATCCAGAGCT |
|  | **DICER R** | AAACGAACCACCAAGTTGCA |
|  | **DICER LOOP F** | CCTCCAGCAGTCCCTAGGAT |
|  | **DICER LOOP R** | CCGGGAACATCACCTTACAC |

| **CUL9** | **CUL9 F** | CGGAGATCCCCACTTTTGT |
| --- | --- | --- |
|  | **CUL9 R** | AGTATTCAGCATCAGGATCACG |
| **UBE4B** | **UBE4B F** | GGACTTGATTGGCCAGATTT |
|  | **UBE4B R** | CTGGATTGGAGCCAGTGTTT |
| **PMSE4** | **PMSE4 F** | CTCGAGTGGATGGAAGGAA |
|  | **PMSE4 R** | AGGCTTGTCAAAGCCACC |
| **CUL4B** | **CUL4B F** | CTTCAACCTCGTCCTTCTGC |
|  | **CUL4B R** | GTTGCAGCAGTTGGTGAAGA |
| **UBR3** | **UBR3 F** | GGTTCGTCCCAAAACTTCAA |
|  | **UBR3 R** | GTCATCAAGCCAAGGAGGAT |
| **UBA6** | **UBA6 F** | ACAATGGGCACTAAGGGACA |
|  | **UBA6 R** | TCTCTTGCCCACTGTATGGT |
| **IKZF1** | **IKZF1 F** | AGAGCAACAACGAGGAGCAG |
|  | **IKZF1 R** | ACGTGATCCAGGAAGAGCAC |
|  | **IKZF1 LOOP F** | CAGTCCCCAGAAGCAGAGA |
|  | **IKZF1 LOOP R** | GACTTTCCCCAACCAATTCA |
| **PSMD1** | **PSMD1 F** | CAAACAATGTGTGGAAAATGC |
|  | **PSMD1 R** | CTTCGTGTCTCCAGAGCAA |
| **CUL5** | **CUL5 F** | gttaacctttgttctaatcctga |
|  | **CUL5 R** | TGTACACCATTTTGTTGTAAATACG |
| **IRF4** | **IRF4 F** | GCTTGGGCACTGTTTAAAGG |
|  | **IRF4 R** | TTGTACGGGTCTGAGATGTCC |
|  | **IRF4 LOOP F** | TTGCACACACTTTCATGCAG |
|  | **IRF4 LOOP R** | TGCTGCCCAATACATATCC |
| **ERAP1** | **ERAP1 F** | GATGGGACACCATTTCCTTG |
|  | **ERAP1 R** | CTCTCTCCAGCTCCCTTCCT |
| **KLF13** | **KLF13 F** | CCGCAGAGGAAGCACAA |
|  | **KLF13 R** | AAGGGCCTCTCACCTGTG |
|  | **KLF13 LOOP F** | AGCAAGAGACCGGAACATTG |
|  | **KLF13 LOOP R** | AGTCTGAGTTGGGGACATGG |
| **PRDM1** | **PRDM1 F** | CCCAAAGAATGTCCCAAAGA |
|  | **PRDM1 R** | GGGCTCCCACGTCTTCTAA |
|  | **PRDM1 LOOP F** | TGCTCAACCCCACTTCTCTC |
|  | **PRDM1 LOOP R** | GCTACAGGCCTTGTCCTTCA |
| **RICTOR** | **RICTOR F** | ACTCCAAATATGTTGACTTGATTG |
|  | **RICTOR R** | CTTTGGTTACTCCGACGAACA |
|  | **RICTOR LOOP F** | AGAAGCAGAGGCTGTGTTGG |
|  | **RICTOR LOOP R** | CCAGTAACTGCGGAACAGT |
| **ATR** | **ATR F** | TGCAGCTTCTGCTCTCATTC |
|  | **ATR R** | GTGTTCTCCAATACCGCAGCA |
|  | **ATR LOOP F** | ATGCTAACAGGTCCGAGTGG |
|  | **ATR LOOP R** | TGGTTTGATGCTATGCTCC |
| **AKAP9** | **AKAP9 F** | AGCCATCATTGCCTCTGAAA |
|  | **AKAP9 R** | TGTTCAGAATCAGGGCTCAG |
| **PCM1** | **PCM1 F** | ATGGAGAGCAGCCAGATTG |
|  | **PCM1 R** | GGAGGCTGAGAATCACGAAG |
| **CENPF** | **CENPF F** | GATCAATTGAAGGAGCTCACA |
|  | **CENPF R** | GCCTGGTGTTTCTTTTCAGC |
|  | **CENPF LOOP F** | GAAAAACAAGGTCAGTTGTCAGAA |
|  | **CENPF LOOP R** | GGGCTCTCAGCTTTTCAATG |
| **XRN1** | **XRN1 F** | GAGATGAATATGGATTACCCTCTCA |
|  | **XRN1 R** | TCAGTTACTGGTGTGGATACAGG |
| **KMT2C** | **KMT2C F** | ATCAACCCAATCGCTCATTC |
|  | **KMT2C R** | CGGCTTGTTGAGGAAGCTCAC |
| **XBP1 3´region** | **XBP1 F** | GTTGGGCATTCTGGACAACT |
|  | **XBP1 R** | CTTCCAGCTTGGCTGATGAC |
| **B2M** | **B2M F** | CTCACGTCATCCAGCAGAGA |
|  | **B2M R** | CGGCAGGCATACTCATCTTT |
| **BLOC1S1** | **BLOC1S1 F** | CCCAATTTGC CAAGCAGACA |
|  | **BLOC1S1 R** | CATCCCCAATTTCCTT GAGTGC |
| **GAPDH** | **GAPDH F** | GGGTGGAATCATATTGGAACATGTA |
|  | **GAPDH R** | CAGGGCTGCTTTTAACTCTGGTAA |
