# Supplementary figures and images for: RNA sequencing identifies novel regulated IRE1-dependent decay targets that affect multiple myeloma survival and proliferation
Source: Exp Hematol Oncol. 2022 Mar 31;11:18. doi: 10.1186/s40164-022-00271-4 (PMC8969279; doi:10.1186/s40164-022-00271-4)

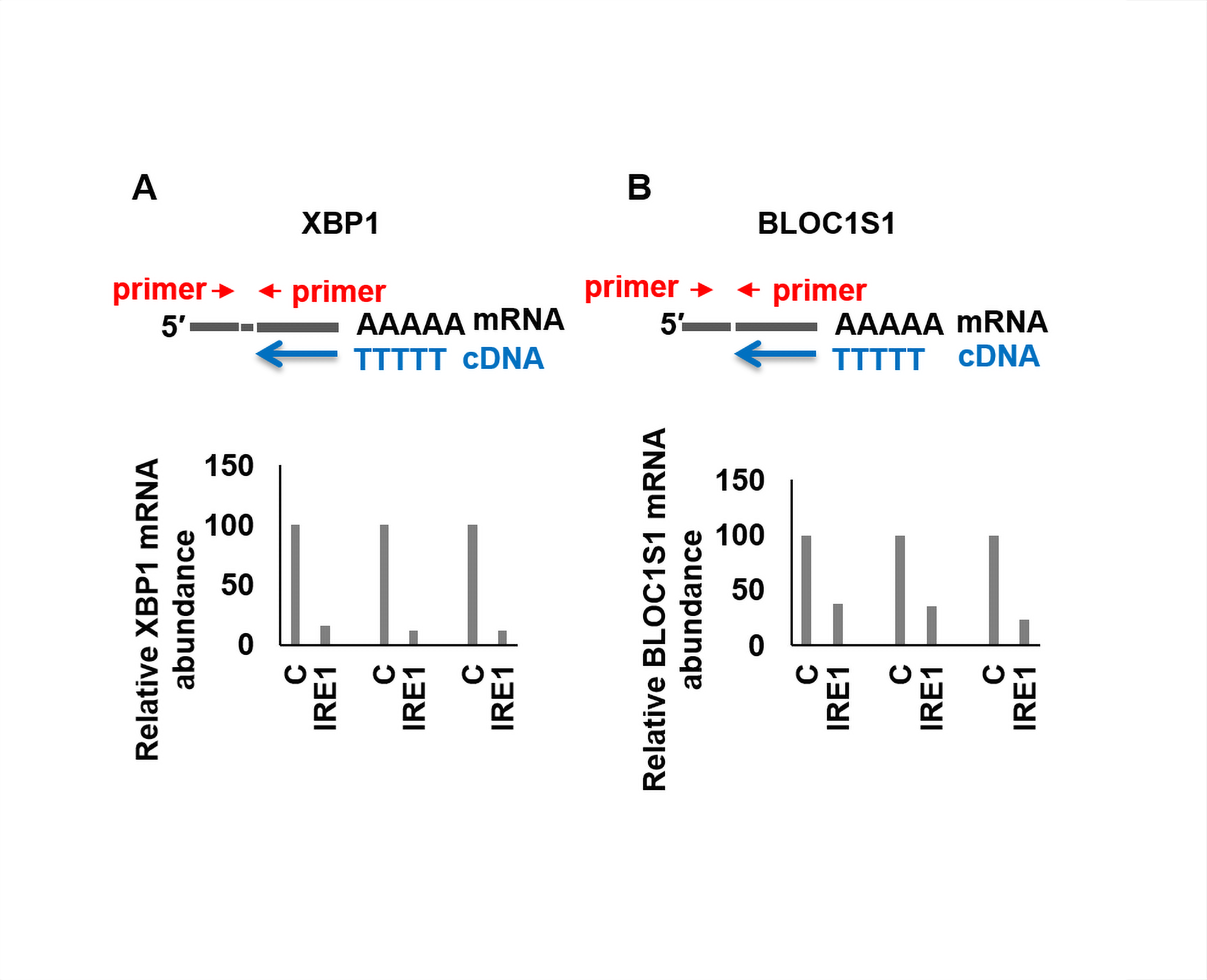

Supplement: Supplementary file 2 — Additional file 2: Fig. S1. Efficiency of cleavage reaction. (A) XBP1 and (B) BLOC1S1 mRNA levels measured by qRT-PCR using the cDNAs synthesized with oligo (dT), and primers mapping the cleavage site of IRE1 from mock and IRE1-treated samples. [file 40164_2022_271_MOESM2_ESM.tif]

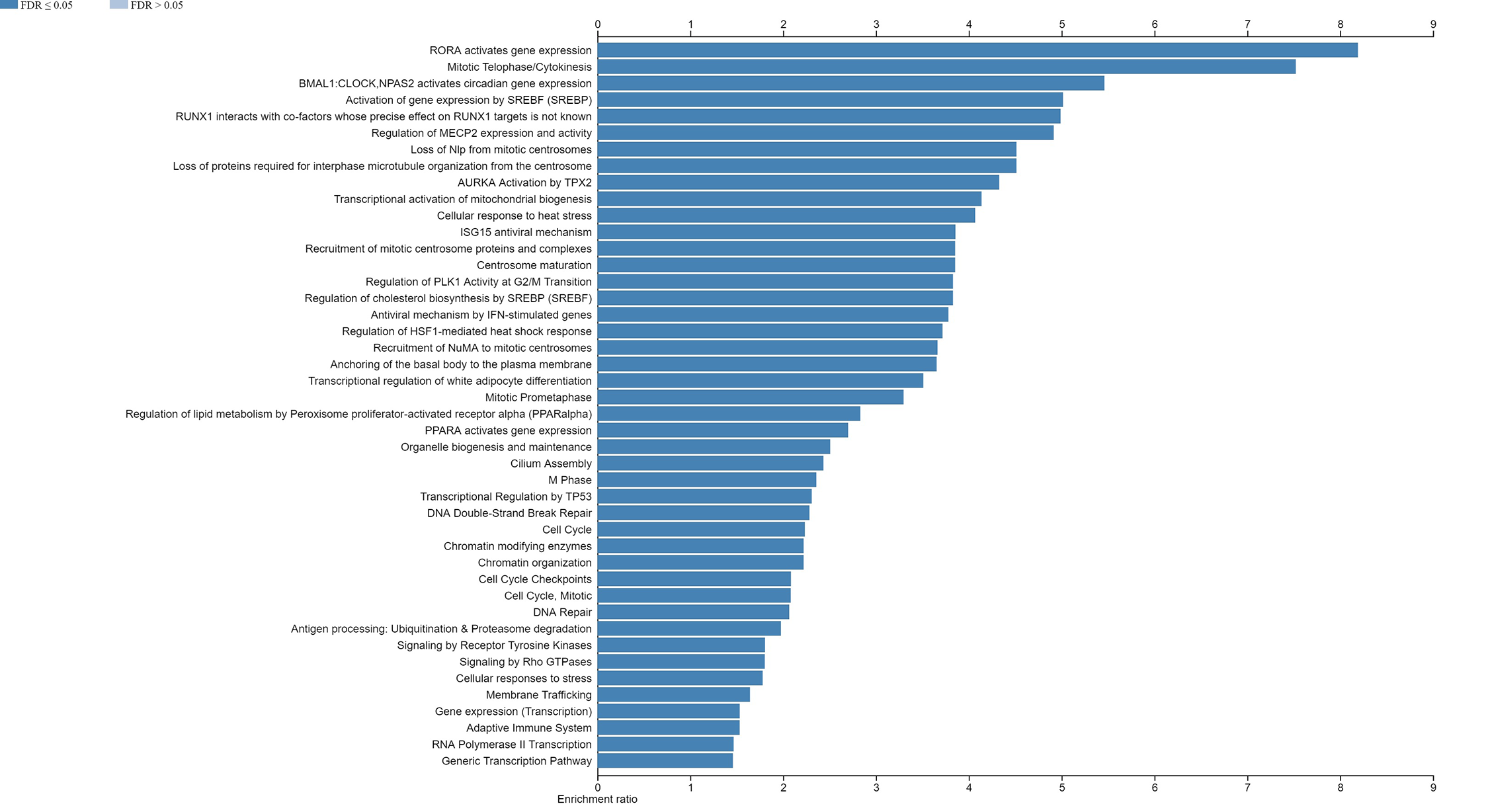

Supplement: Supplementary file 4 — Additional file 4: Fig. S2. Reactome pathway analysis using RNA-seq data. Bar chart representing the most significantly enriched pathways. FDR ≤ 0.05. [file 40164_2022_271_MOESM4_ESM.tif]

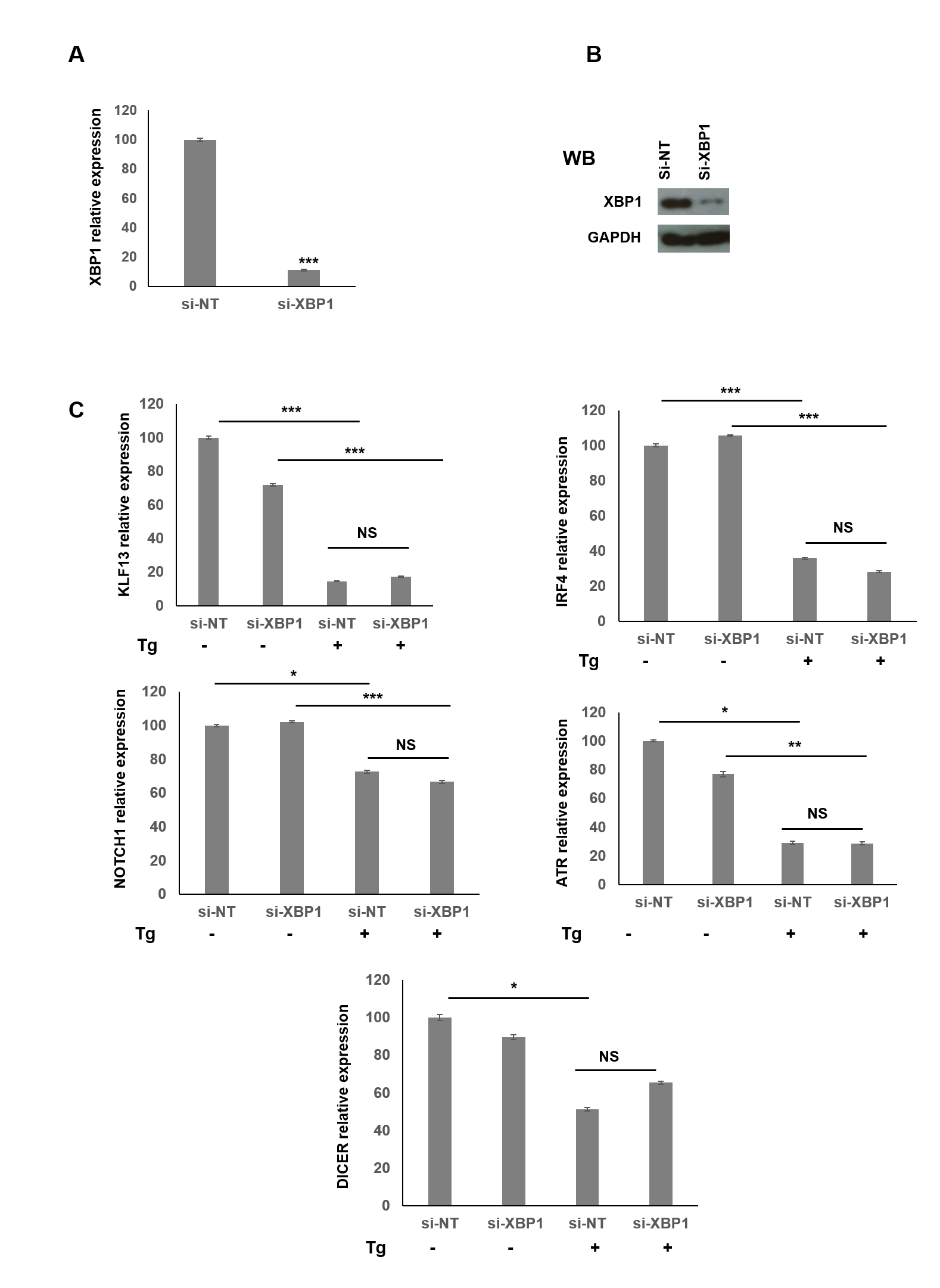

Supplement: Supplementary file 6 — Additional file 6: Fig. S4. XBP1 knockdown. (A) mRNA levels of XBP1 in H929 determined by qRT-PCR 48 h after transfection with XBP1 siRNA. (B) Western blot of XBP1 in H929. (C) mRNA levels of the indicated genes in XBP1 knockdown cells determined by qRT-PCR. H929 cells were treated in the presence or absence of thapsigargin. All results are presented as the means ± SD of three experiments. (*p < 0.05, **p < 0.01, ***p < 0.001). “NS” indicates not significant (p > 0.05). [file 40164_2022_271_MOESM6_ESM.tif]

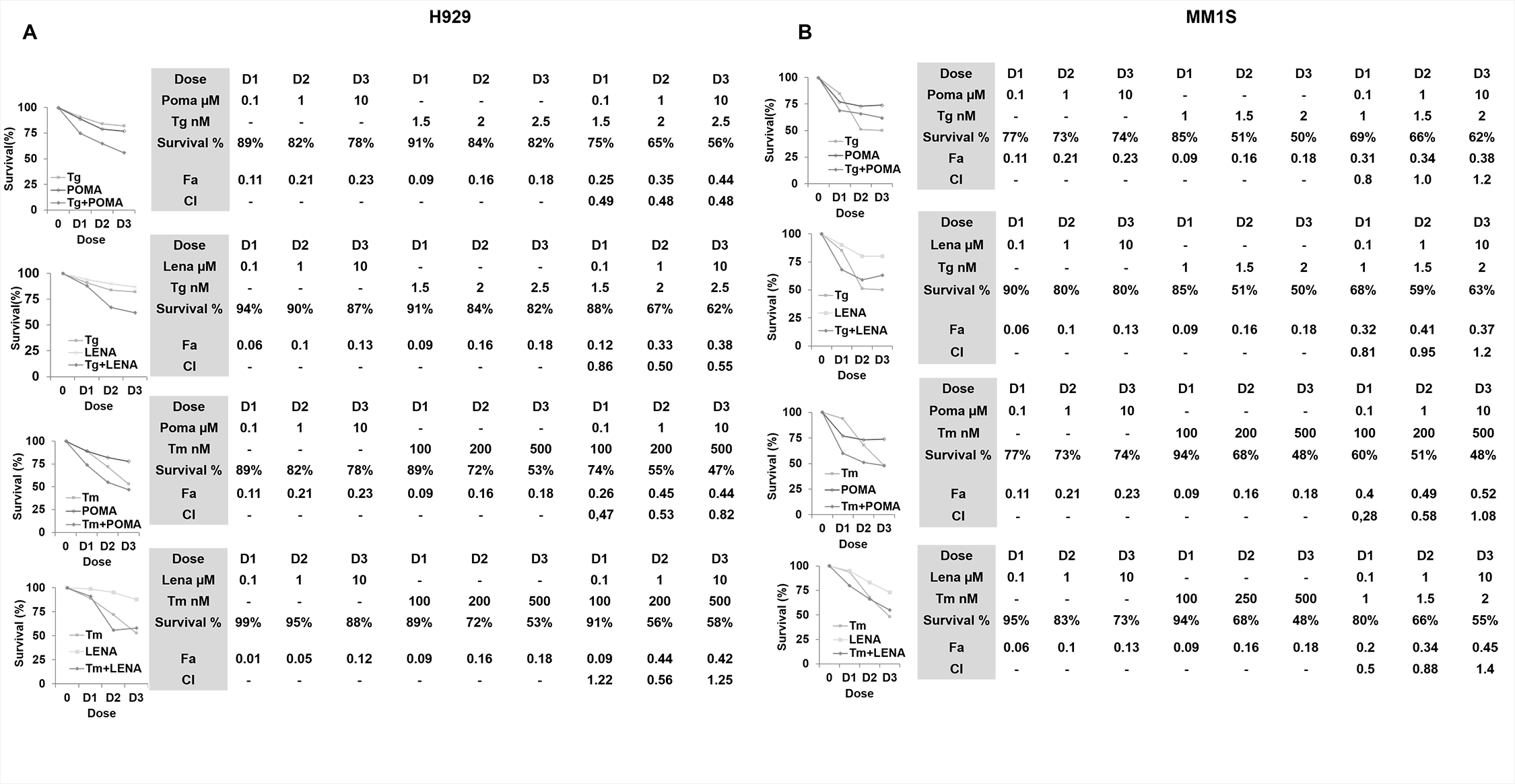

Supplement: Supplementary file 7 — Additional file 7: Fig. S5. Synergistic effect of ER-stress inducers and IMiDs treatment in MMCLs. (A) H929 and (B) MM1S cells were exposed for 48 h to the indicated concentrations of ER- stress inducers and IMiDs, and cell viability assay was assessed by MTT. CI values less than 1 indicated a synergistic effect. These values were calculated using Compusyn Software. C: control (untreated cells). IMiDs; poma (pomalidomide) or lena (lenalidomide). ER inducers; Tm (tunicamycin) or Tg (thapsigargin). [file 40164_2022_271_MOESM7_ESM.tif]
