# Supplementary material for: RNA sequencing identifies novel regulated IRE1-dependent decay targets that affect multiple myeloma survival and proliferation
Source: Exp Hematol Oncol. 2022 Mar 31;11:18. doi: 10.1186/s40164-022-00271-4 (PMC8969279; doi:10.1186/s40164-022-00271-4)
Supplement: Supplementary file 5 — Additional file 5: Fig. S3. Validation of putative IRE1 substrates. Exon-usage plots of the 28 remaining putative mRNAs, showing the number of reads in mock (red) and IRE1-treated (blue) samples. The black arrows represent the site of primers used in the 5´ region of the putative IRE1-substrates. Red arrows represent the site of primers mapping the predicted cleavage site. Right panel of each exon-usage plot shows the abundance of mRNA in the corresponding target. All results are presented as the means ± SD of three experiments. (*p < 0.05, **p < 0.01, ***p < 0.001). [file 40164_2022_271_MOESM5_ESM.pdf]

ATM

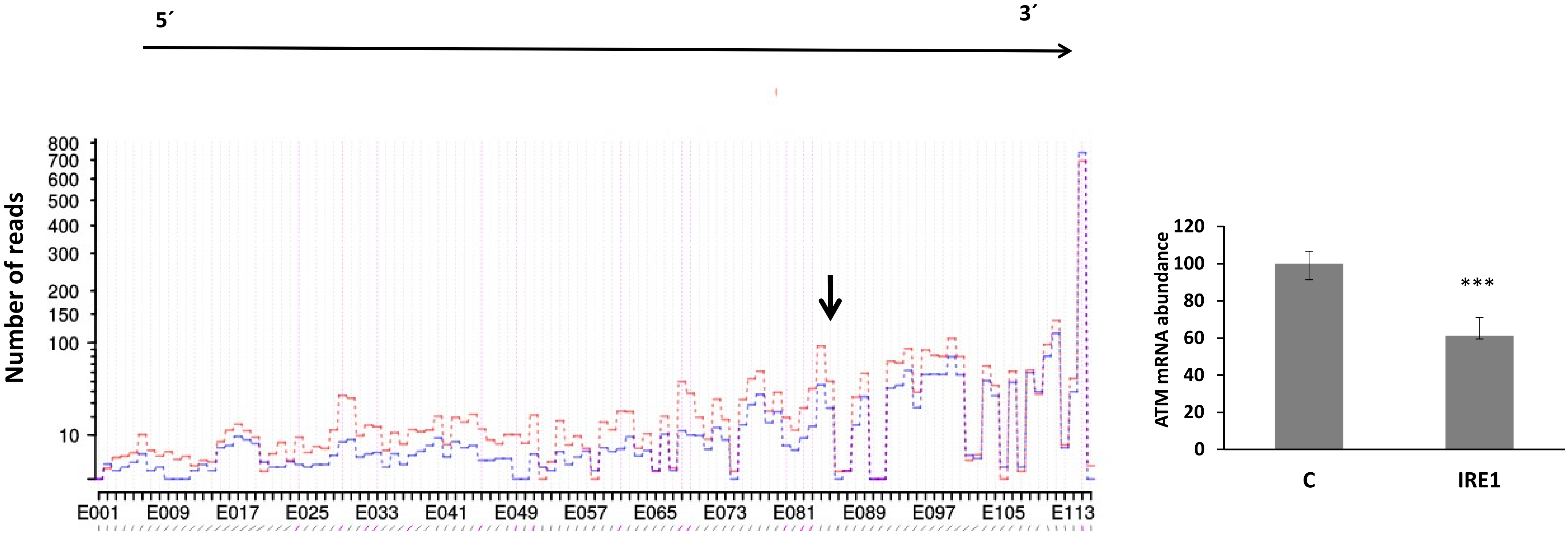

5´
3´
Number of reads
800
700
600
500
400
300
200
150
100
10
E001
E009
E017
E025
E033
E041
E049
E057
E065
E073
E081
E089
E097
E105
E113
ATM mRNA abundance
120
100
80
60
40
20
0
***
C
IRE1

VPS13C

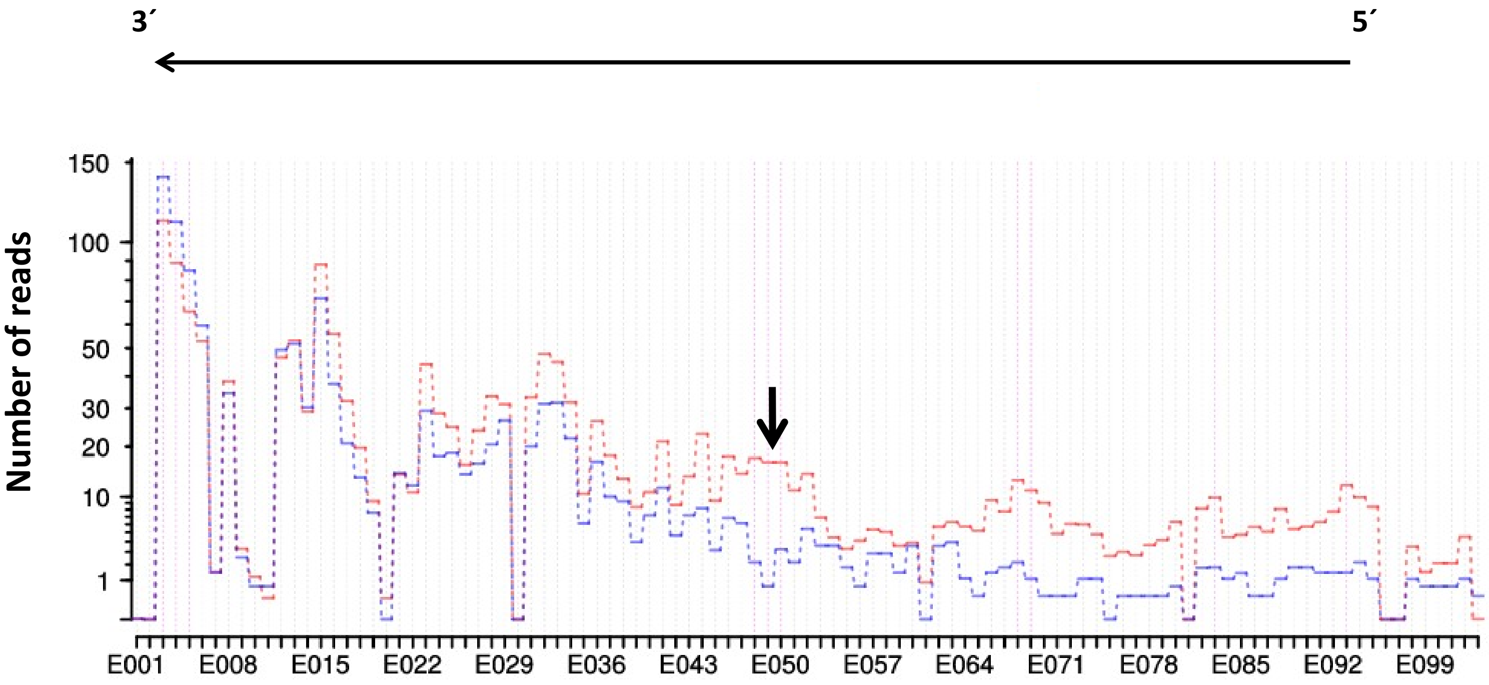

3´
5´
Number of reads
150
100
50
30
20
10
1
E001
E008
E015
E022
E029
E036
E043
E050
E057
E064
E071
E078
E085
E092
E099

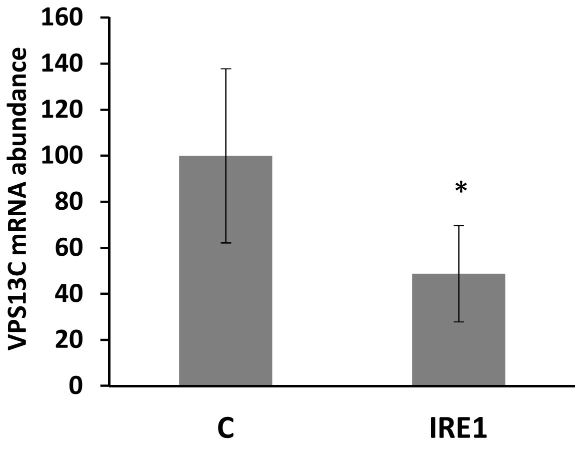

VPS13C mRNA abundance
160
140
120
100
80
60
40
20
0
*
C
IRE1

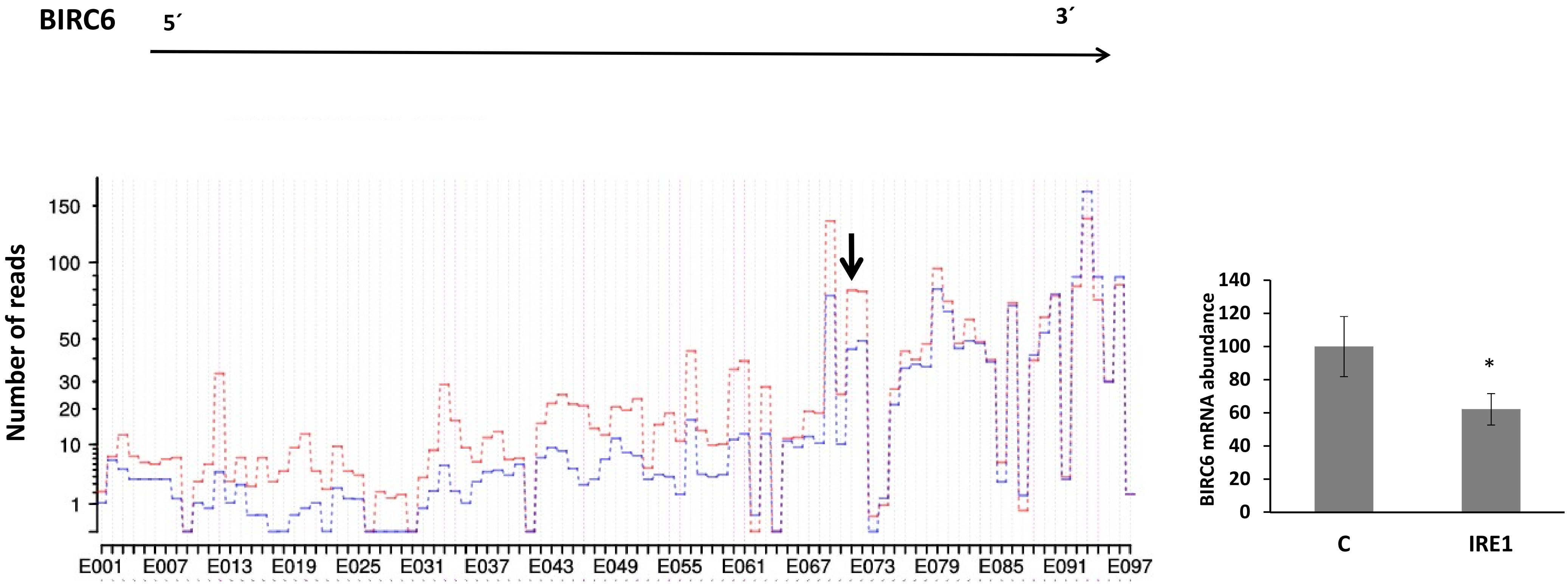
BIRC6
5´
3´
Number of reads
150
100
50
30
20
10
1
E001
E007
E013
E019
E025
E031
E037
E043
E049
E055
E061
E067
E073
E079
E085
E091
E097
BIRC6 mRNA abundance
140
120
100
80
60
40
20
0
*
C
IRE1

KMT2C

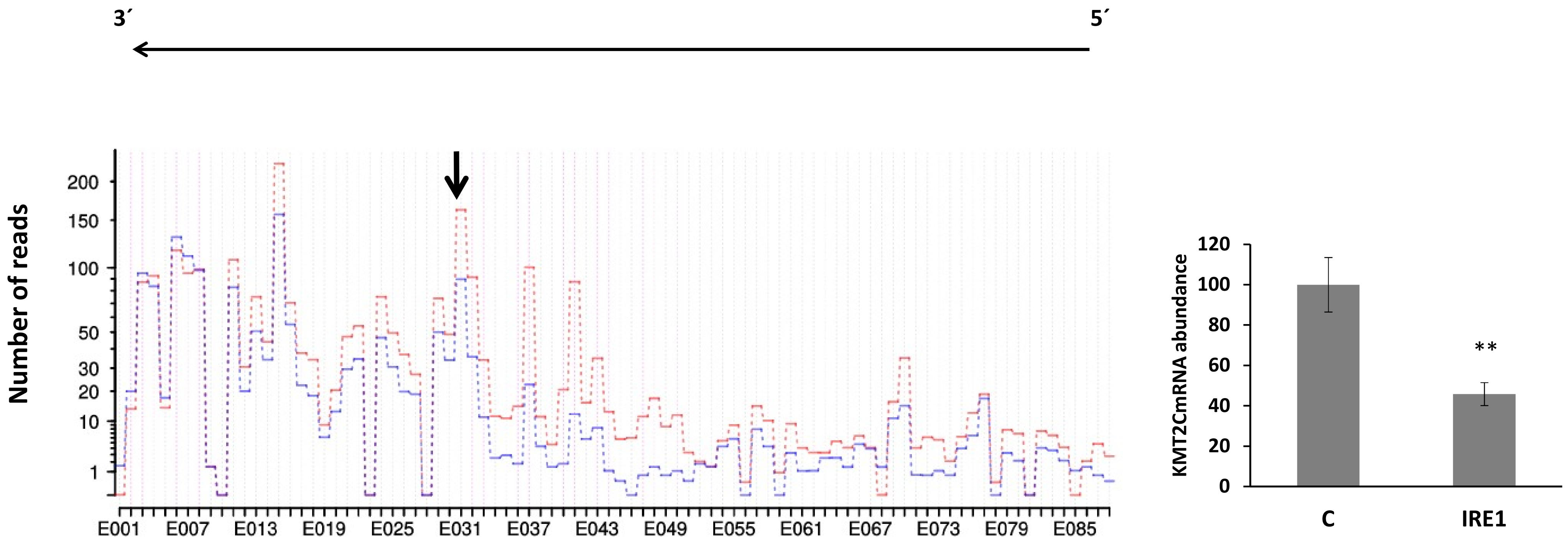

3´
5´
Number of reads
200
150
100
50
30
20
10
1
E001
E007
E013
E019
E025
E031
E037
E043
E049
E055
E061
E067
E073
E079
E085
KMT2CmRNA abundance
120
100
80
60
40
20
0
**
C
IRE1

HUWE1

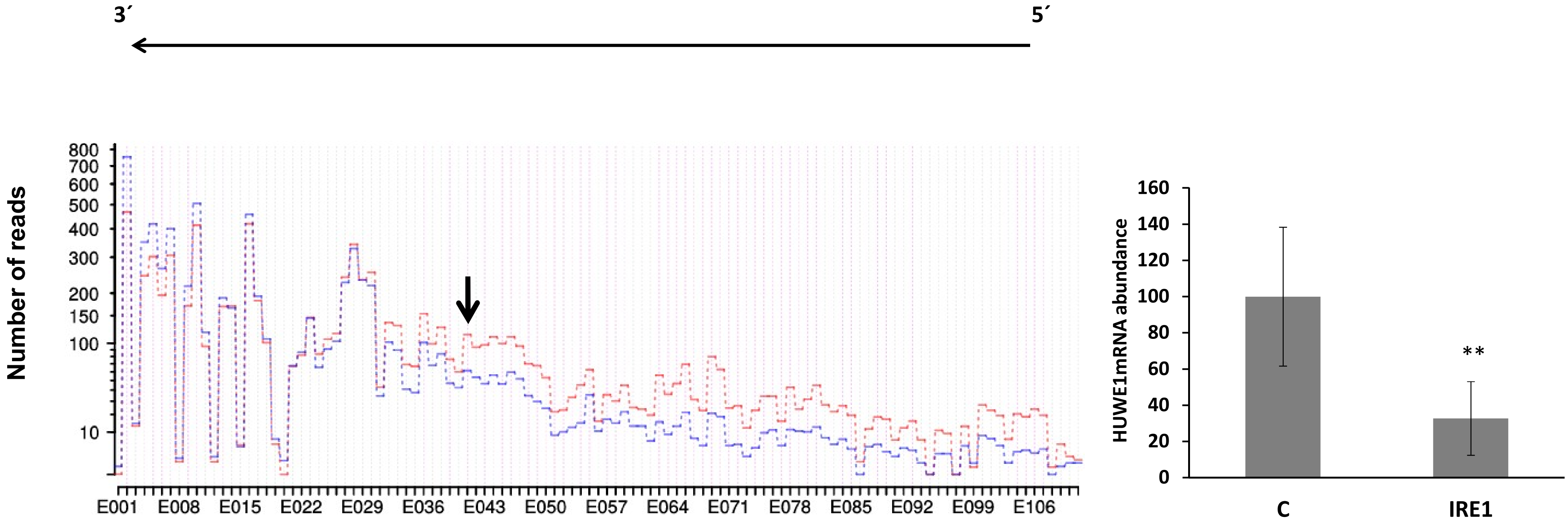

3´
5´
Number of reads
800
700
600
500
400
300
200
150
100
10
E001
E008
E015
E022
E029
E036
E043
E050
E057
E064
E071
E078
E085
E092
E099
E106
HUWE1mRNA abundance
160
140
120
100
80
60
40
20
0
**
C
IRE1

CDK12

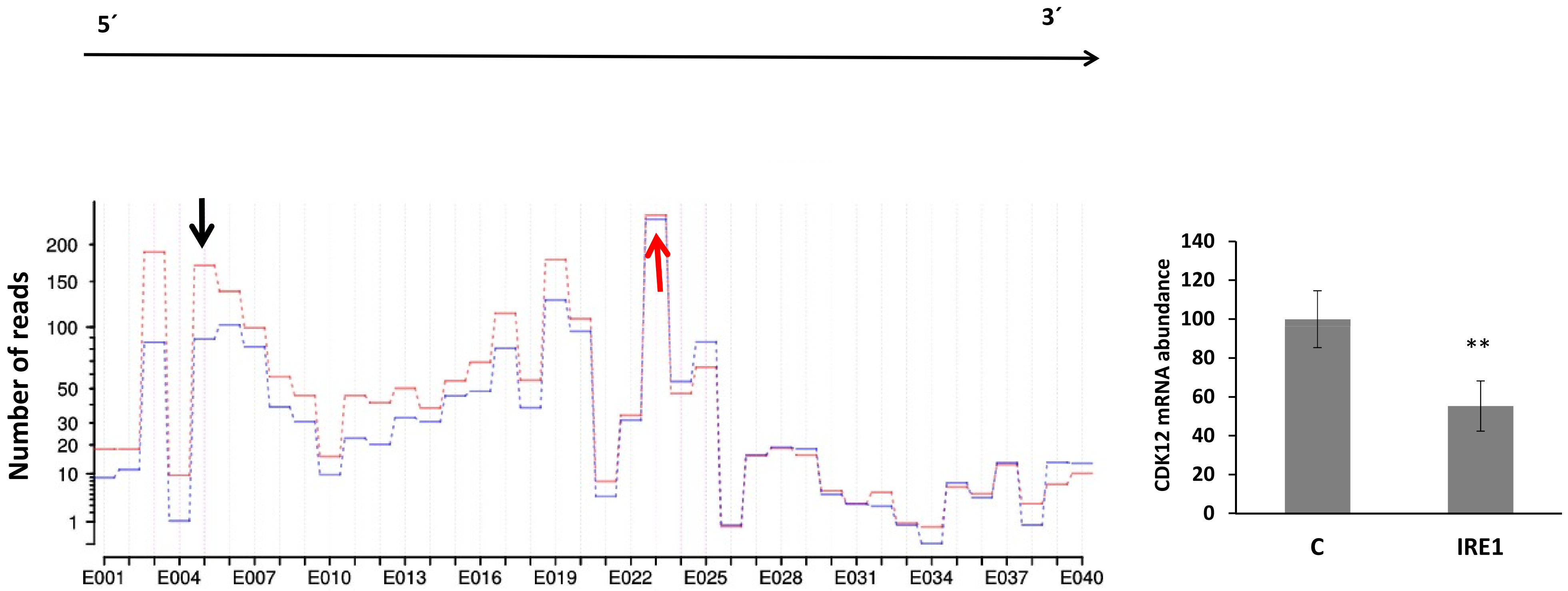

5´
3´
Number of reads
200
150
100
50
30
20
10
1
E001
E004
E007
E010
E013
E016
E019
E022
E025
E028
E031
E034
E037
E040
CDK12 mRNA abundance
140
120
100
80
60
40
20
0
**
C
IRE1

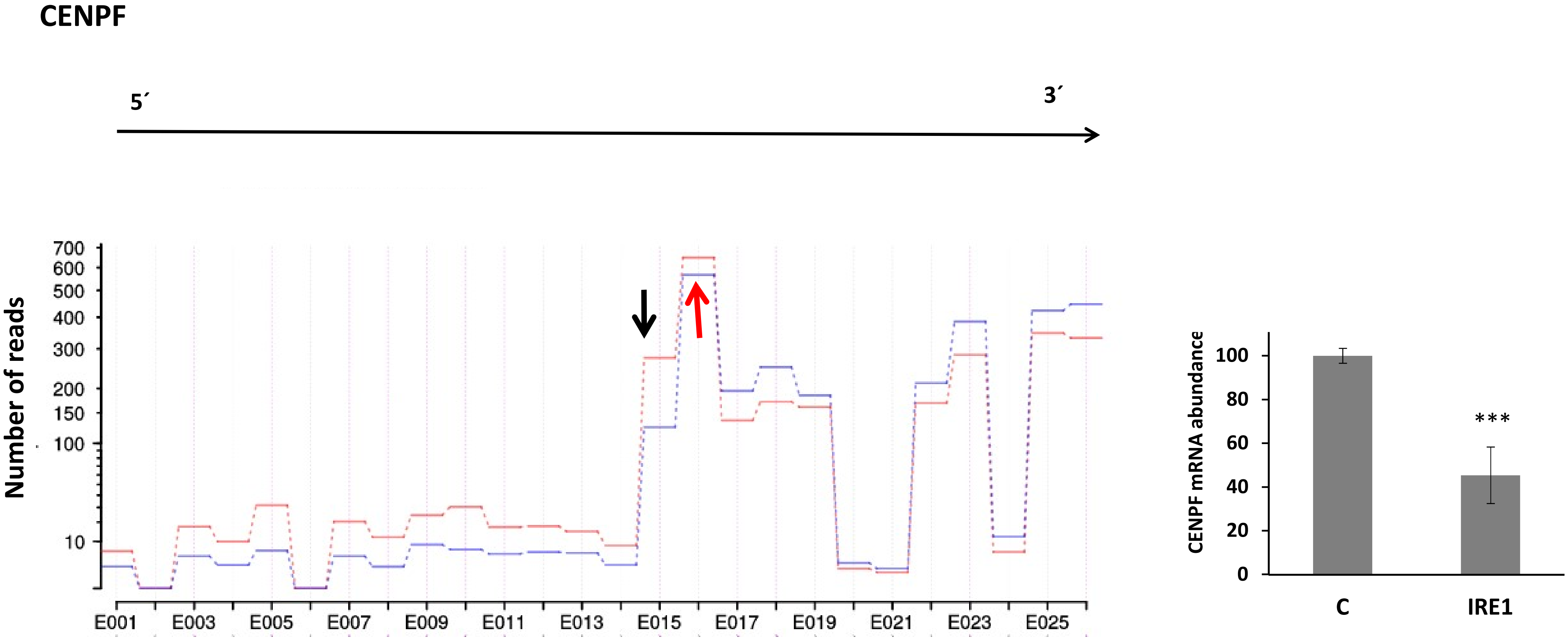
CENPF
5´
3´
Number of reads
700
600
500
400
300
200
150
100
10
E001
E003
E005
E007
E009
E011
E013
E015
E017
E019
E021
E023
E025
CENPF mRNA abundance
100
80
60
40
20
0
***
C
IRE1

VPS13D

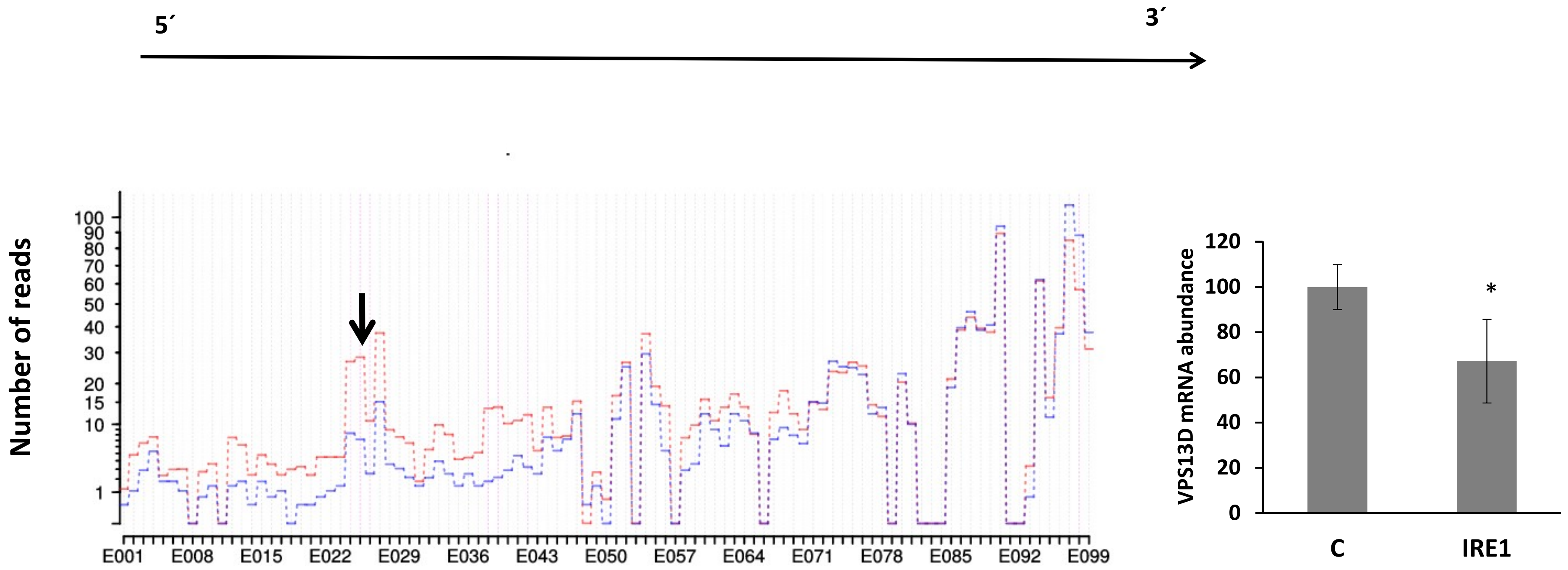

5´
3´
Number of reads
100
90
80
70
60
50
40
30
20
15
10
1
E001
E008
E015
E022
E029
E036
E043
E050
E057
E064
E071
E078
E085
E092
E099
VPS13D mRNA abundance
120
100
80
60
40
20
0
*
C
IRE1

GOLGB1

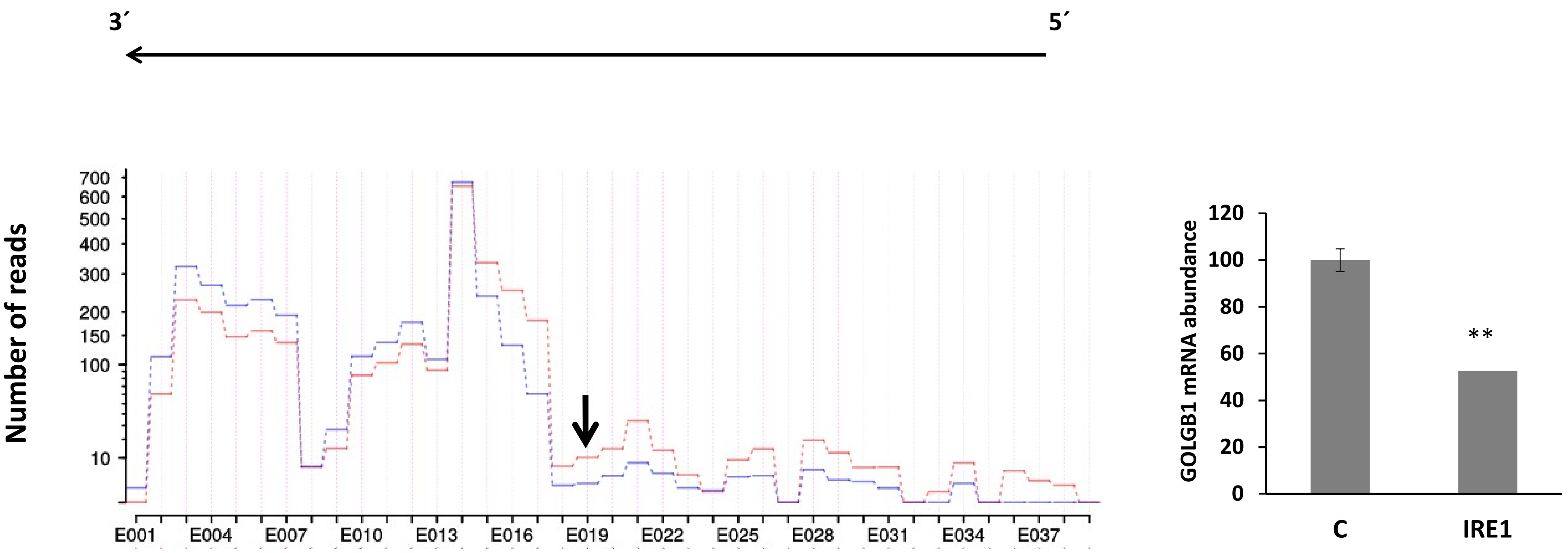

3´
5´
Number of reads
700
600
500
400
300
200
150
100
10
E001
E004
E007
E010
E013
E016
E019
E022
E025
E028
E031
E034
E037
GOLGB1 mRNA abundance
120
100
80
60
40
20
0
**
C
IRE1

FAM168B

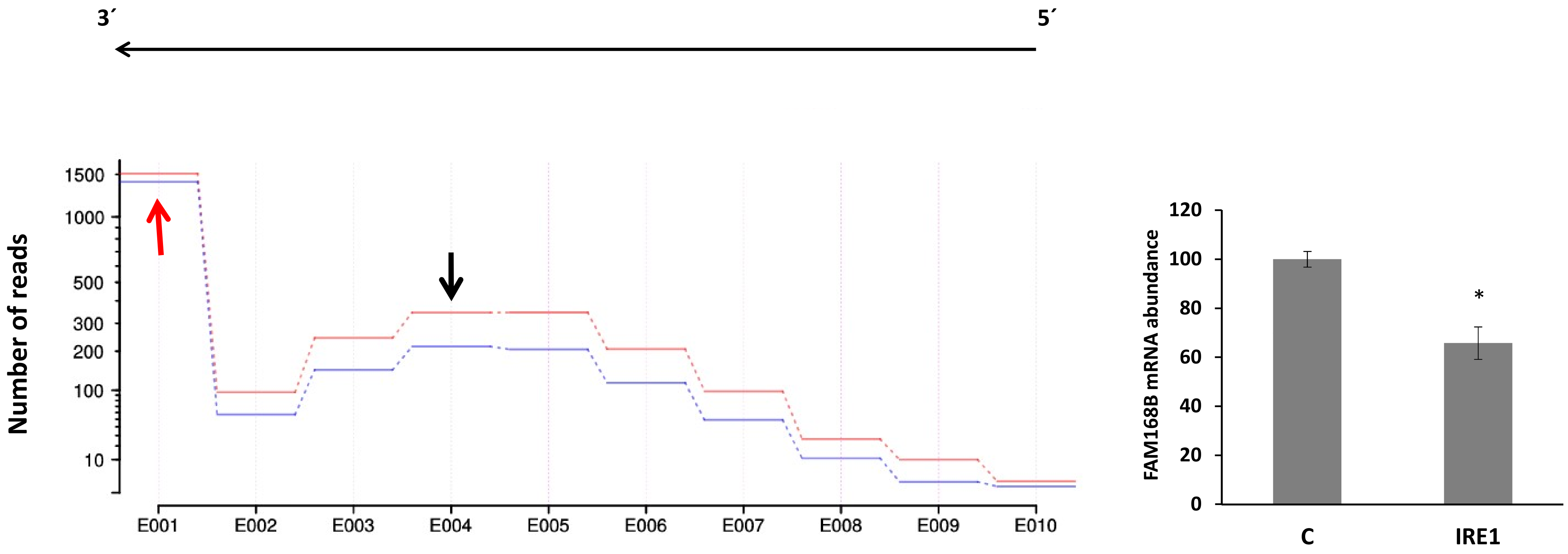
3´
5´
Number of reads
1500
1000
500
300
200
100
10
E001
E002
E003
E004
E005
E006
E007
E008
E009
E010
FAM168B mRNA abundance
120
100
80
60
40
20
0
*
C
IRE1

AKAP9

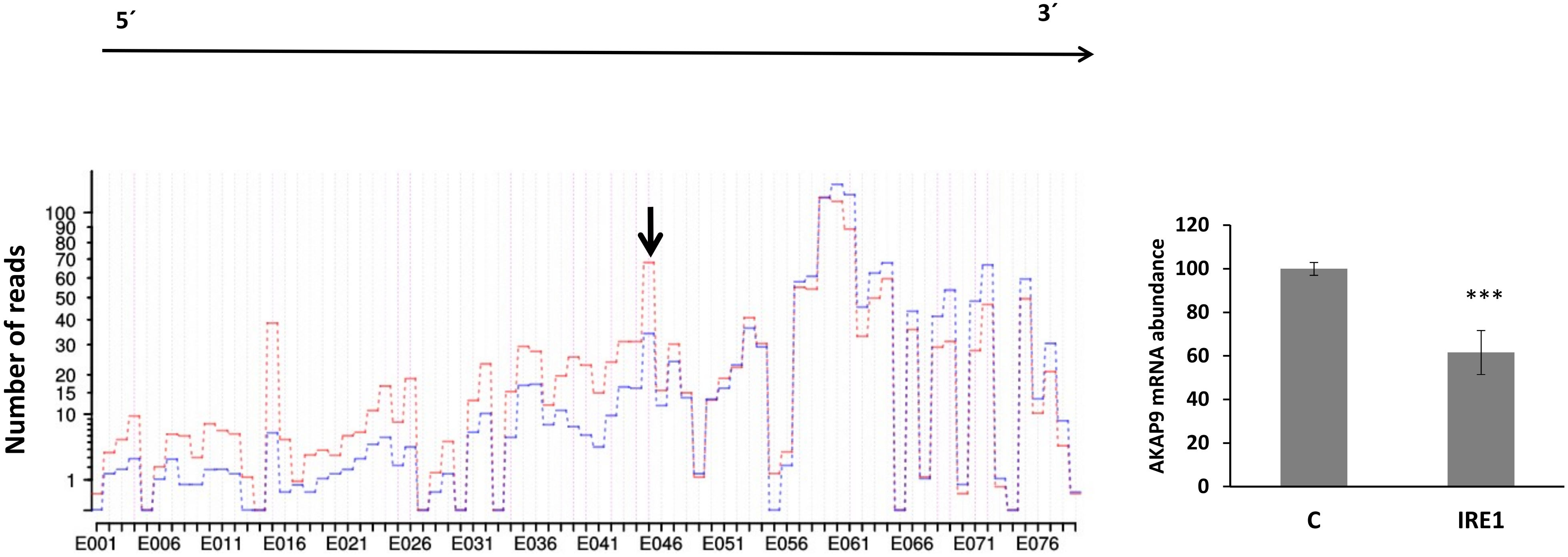

5´
3´
Number of reads
100
90
80
70
60
50
40
30
20
15
10
1
E001
E006
E011
E016
E021
E026
E031
E036
E041
E046
E051
E056
E061
E066
E071
E076
AKAP9 mRNA abundance
120
100
80
60
40
20
0
***
C
IRE1

NOTCH1

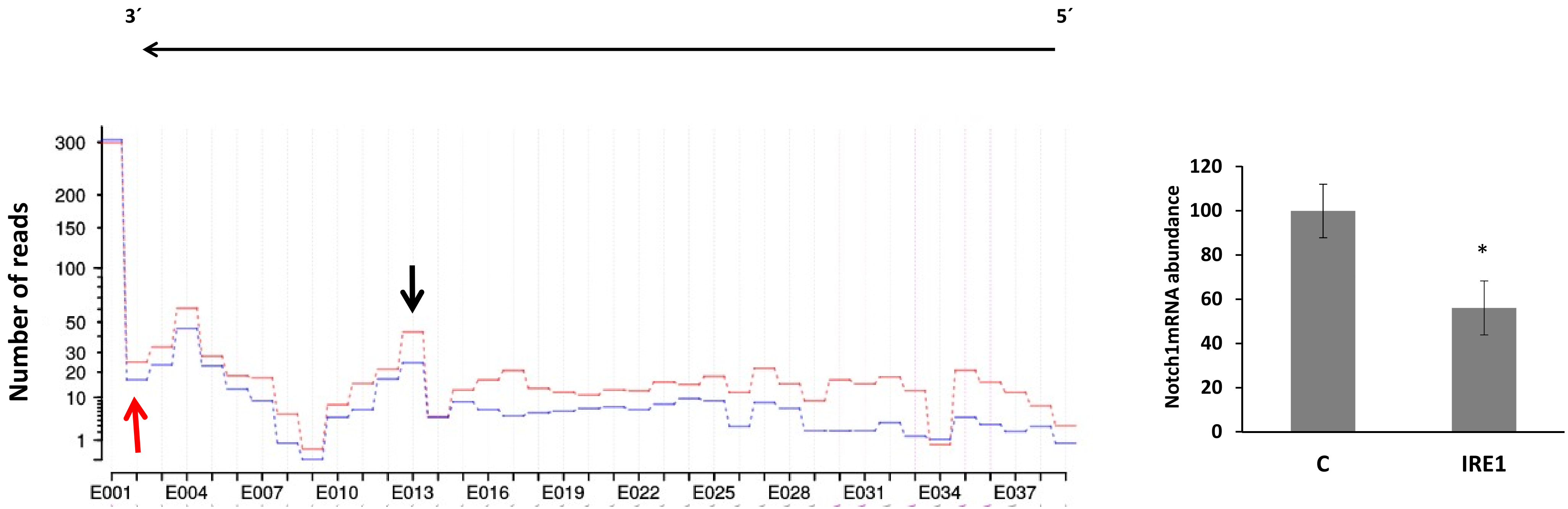

3´
5´
Number of reads
300
200
150
100
50
30
20
10
1
E001
E004
E007
E010
E013
E016
E019
E022
E025
E028
E031
E034
E037
Notch1mRNA abundance
120
100
80
60
40
20
0
*
C
IRE1

PCM1

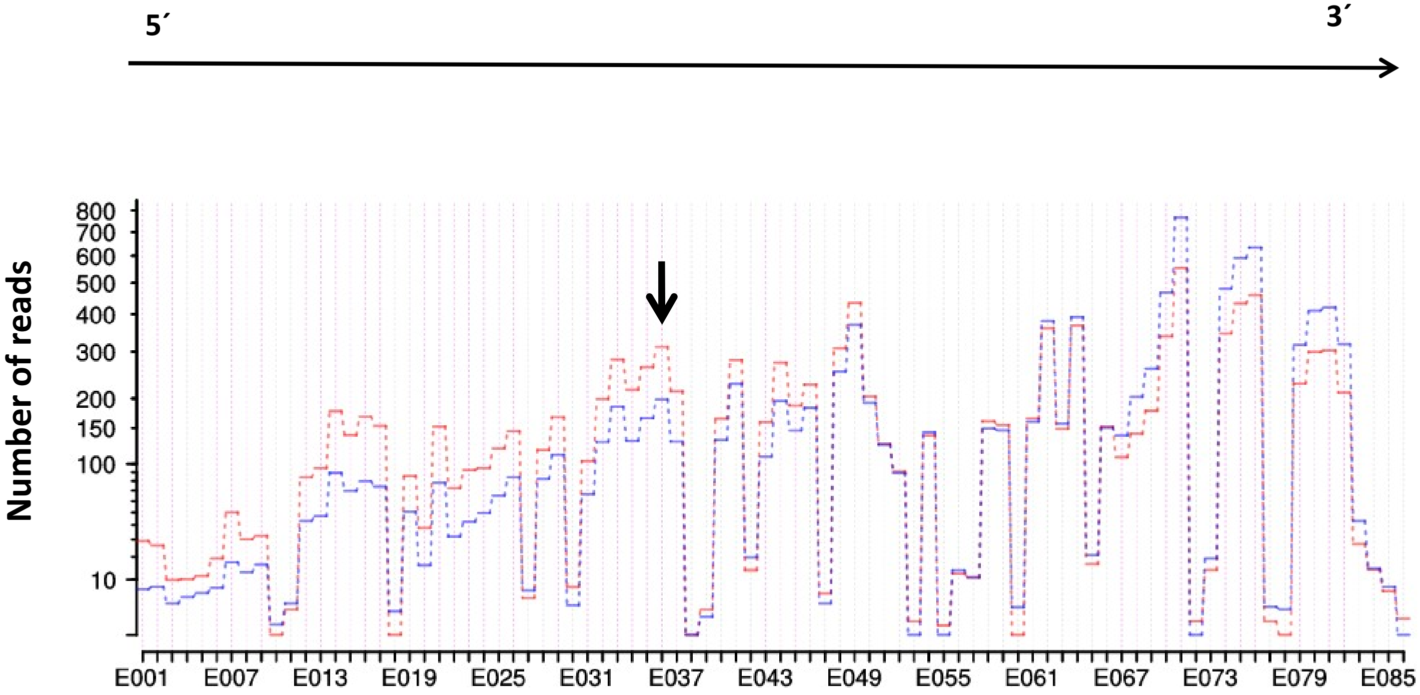

5´
3´
Number of reads
800
700
600
500
400
300
200
150
100
10
E001
E007
E013
E019
E025
E031
E037
E043
E049
E055
E061
E067
E073
E079
E085

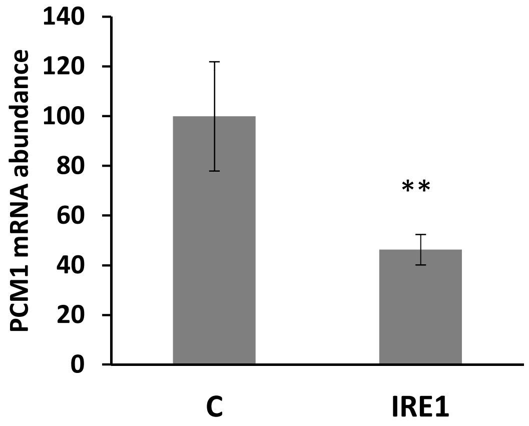

PCM1 mRNA abundance
140
120
100
80
60
40
20
0
**
C
IRE1

UBR2

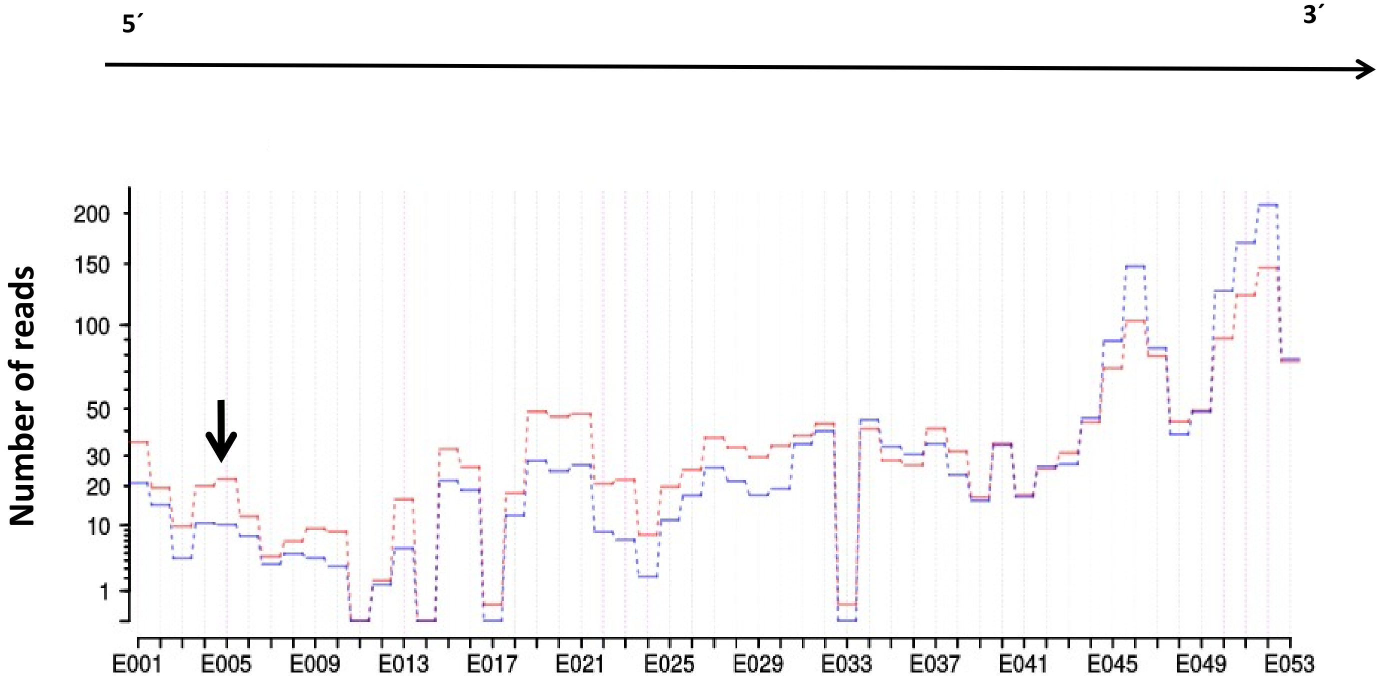
5´
3´
Number of reads
200
150
100
50
30
20
10
1
E001
E005
E009
E013
E017
E021
E025
E029
E033
E037
E041
E045
E049
E053

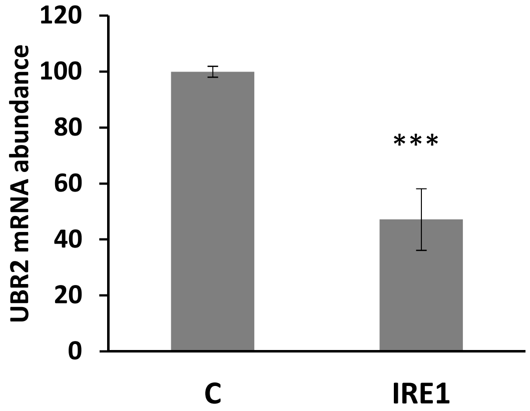
UBR2 mRNA abundance
120
100
80
60
40
20
0
***
C
IRE1

mTOR

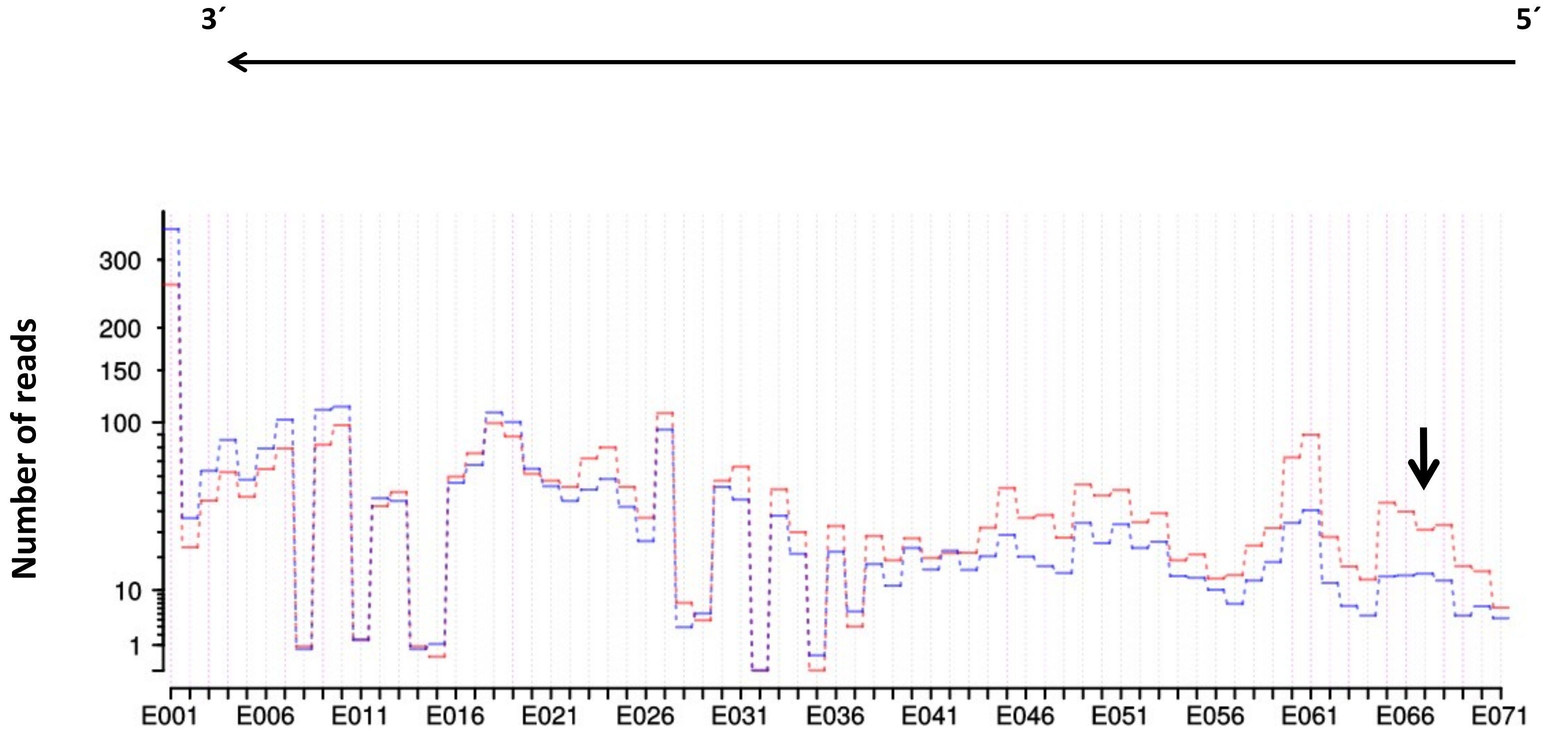

3´
5´
Number of reads
300
200
150
100
10
1
E001
E006
E011
E016
E021
E026
E031
E036
E041
E046
E051
E056
E061
E066
E071

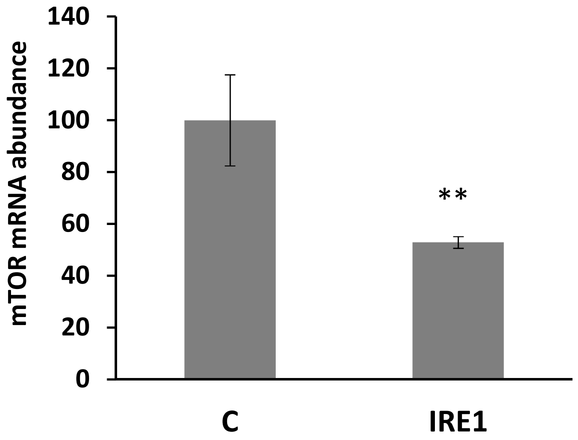

mTOR mRNA abundance
140
120
100
80
60
40
20
0
**
C
IRE1

DICER

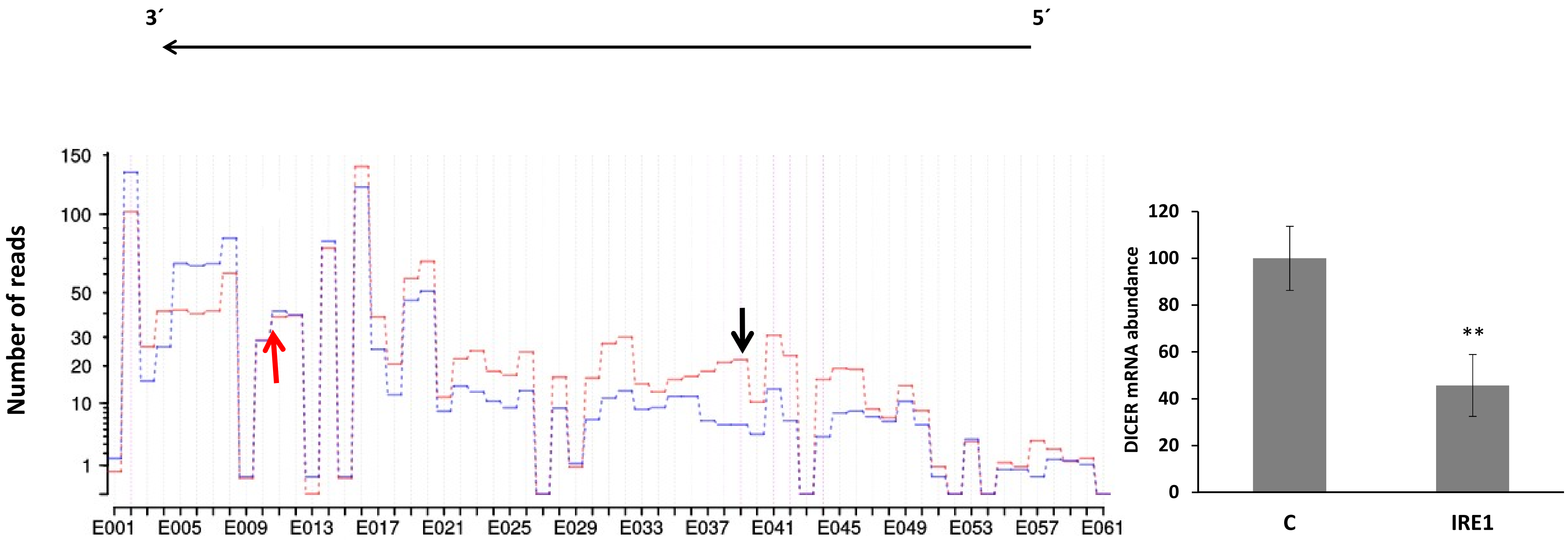
3´
5´
Number of reads
150
100
50
30
20
10
1
E001
E005
E009
E013
E017
E021
E025
E029
E033
E037
E041
E045
E049
E053
E057
E061
DICER mRNA abundance
120
100
80
60
40
20
0
**
C
IRE1

XRN1

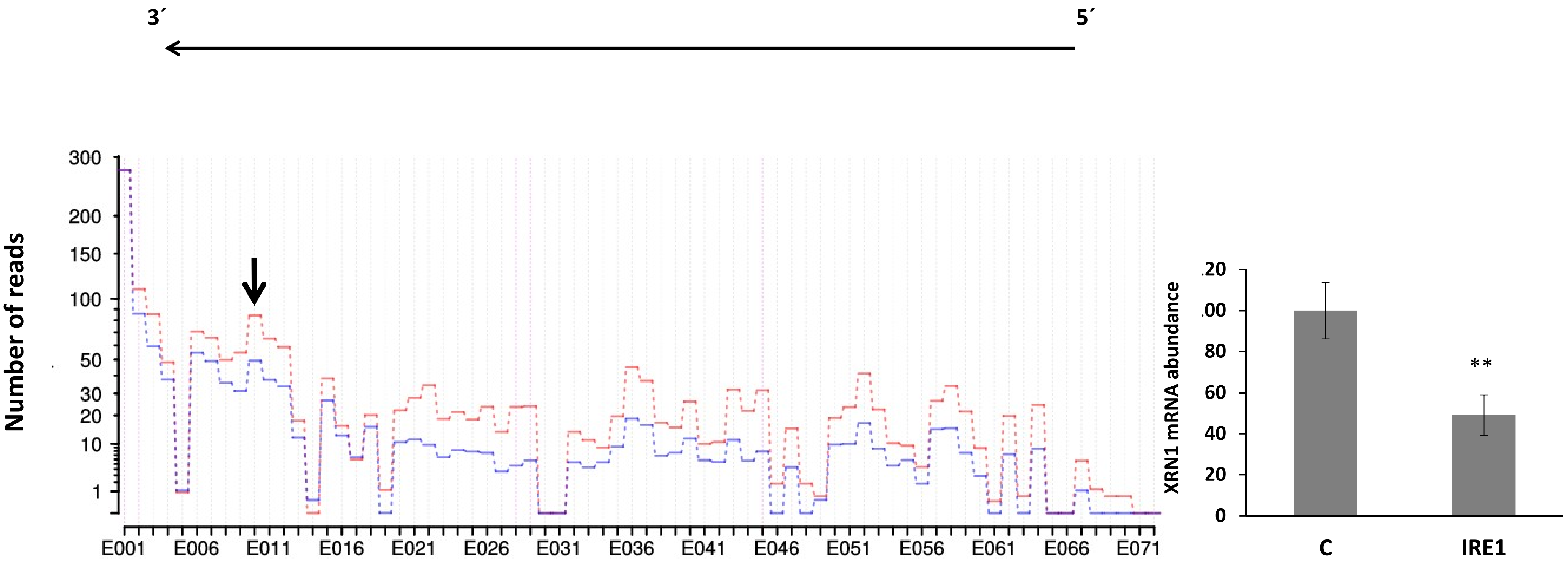
3´
5´
Number of reads
300
200
150
100
50
30
20
10
1
E001
E006
E011
E016
E021
E026
E031
E036
E041
E046
E051
E056
E061
E066
E071
XRN1 mRNA abundance
.20
.00
80
60
40
20
0
**
C
IRE1

CUL9

5´ → 3´

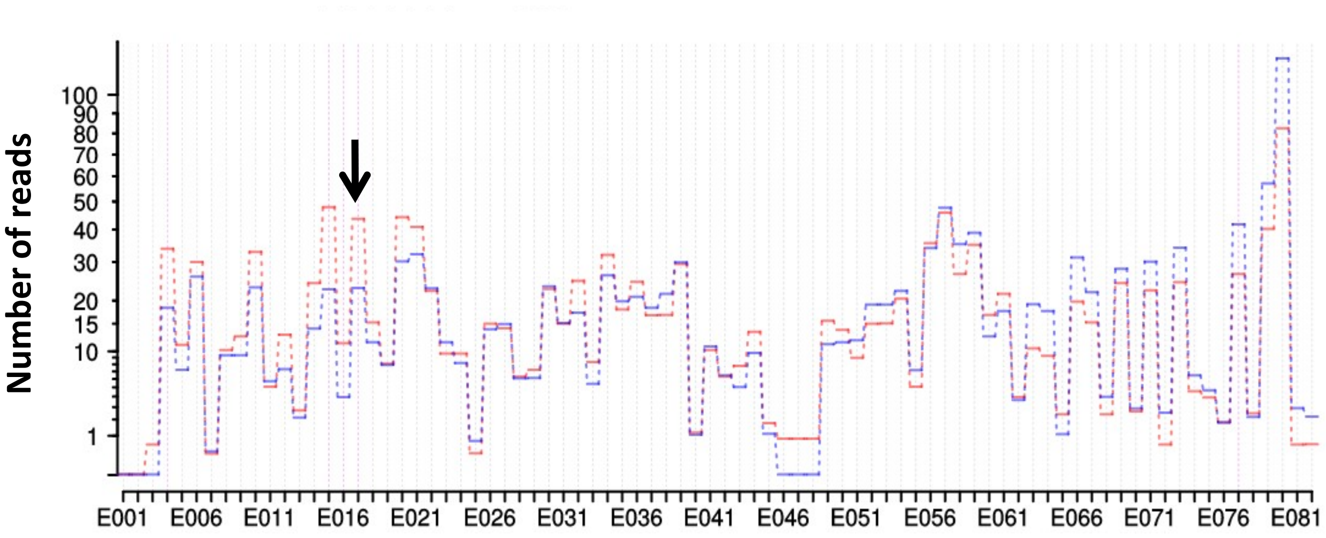
Number of reads
100
90
80
70
60
50
40
30
20
15
10
1
E001
E006
E011
E016
E021
E026
E031
E036
E041
E046
E051
E056
E061
E066
E071
E076
E081

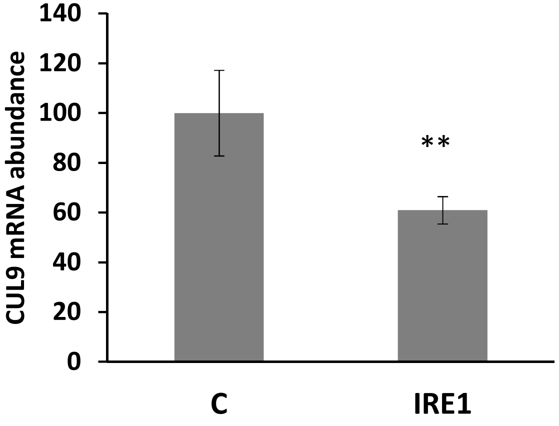
CUL9 mRNA abundance
140
120
100
80
60
40
20
0
**
C
IRE1

UBE4B

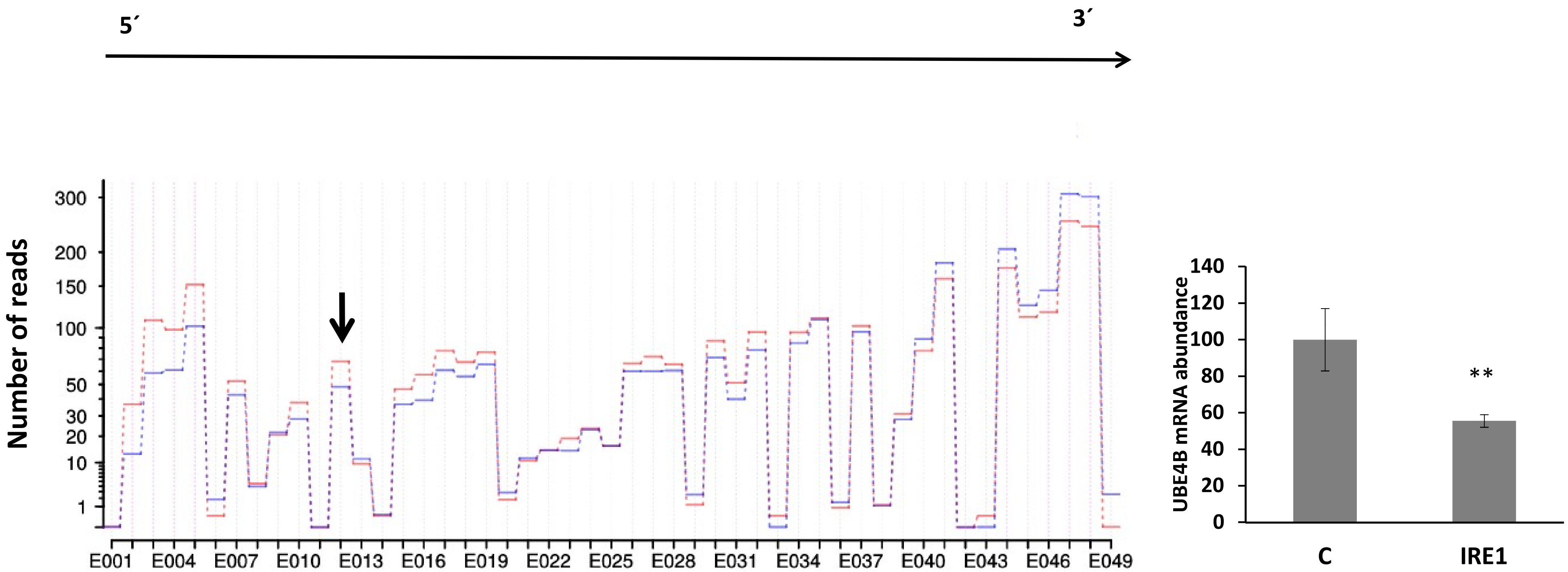
5´
3´
Number of reads
300
200
150
100
50
30
20
10
1
E001
E004
E007
E010
E013
E016
E019
E022
E025
E028
E031
E034
E037
E040
E043
E046
E049
UBE4B mRNA abundance
140
120
100
80
60
40
20
0
**
C
IRE1

PSME4

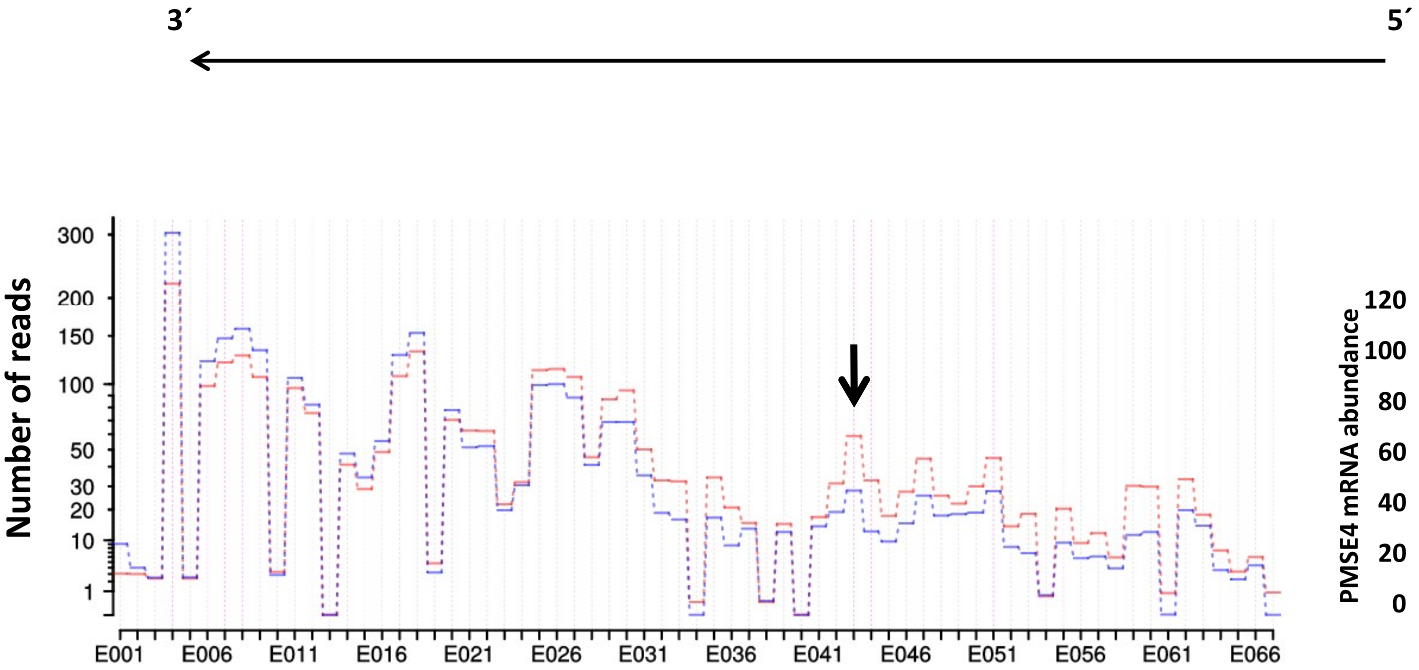
3´
5´
Number of reads
300
200
150
100
50
30
20
10
1
E001
E006
E011
E016
E021
E026
E031
E036
E041
E046
E051
E056
E061
E066

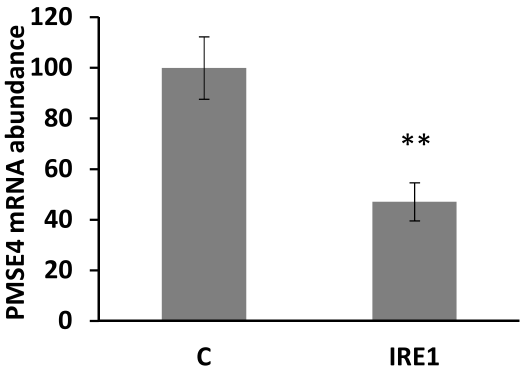
PMSE4 mRNA abundance
120
100
80
60
40
20
0
**
C
IRE1

CUL4B

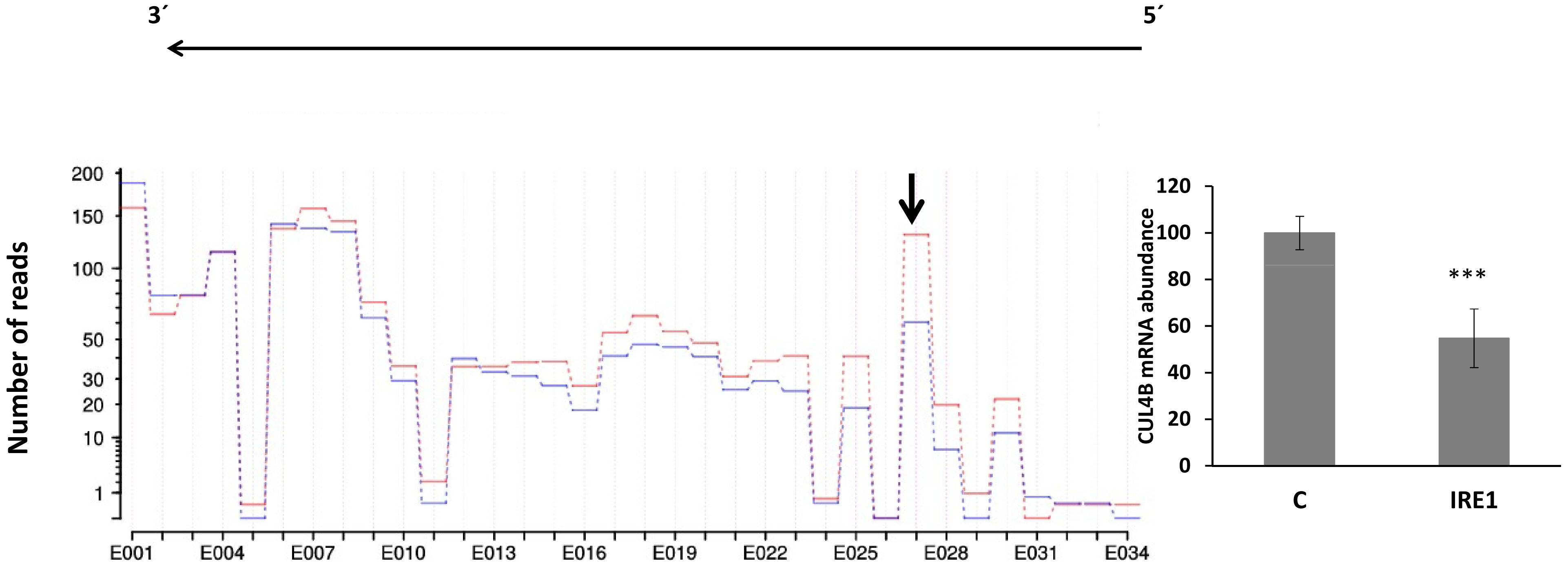

3´
5´
Number of reads
200
150
100
50
30
20
10
1
E001
E004
E007
E010
E013
E016
E019
E022
E025
E028
E031
E034
CUL4B mRNA abundance
120
100
80
60
40
20
0
***
C
IRE1

UBR3

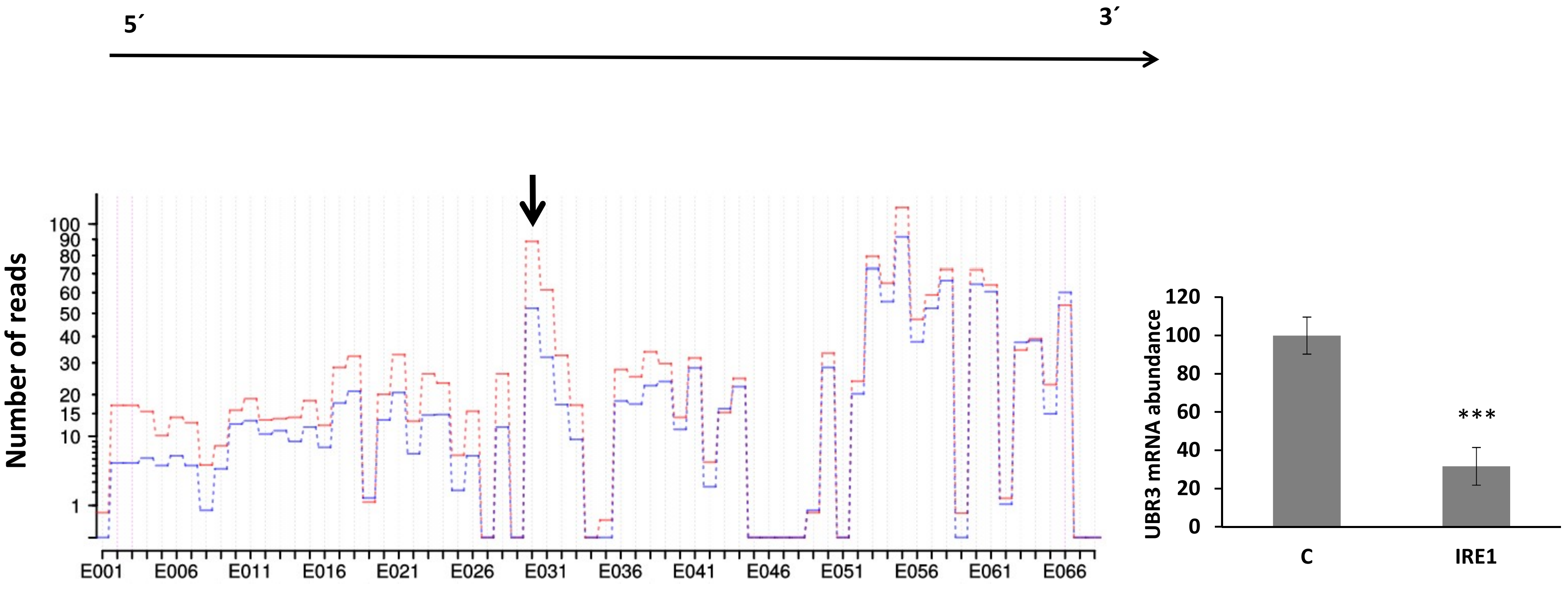
5´
3´
Number of reads
100
90
80
70
60
50
40
30
20
15
10
1
E001
E006
E011
E016
E021
E026
E031
E036
E041
E046
E051
E056
E061
E066
UBR3 mRNA abundance
120
100
80
60
40
20
0
***
C
IRE1

UBA6

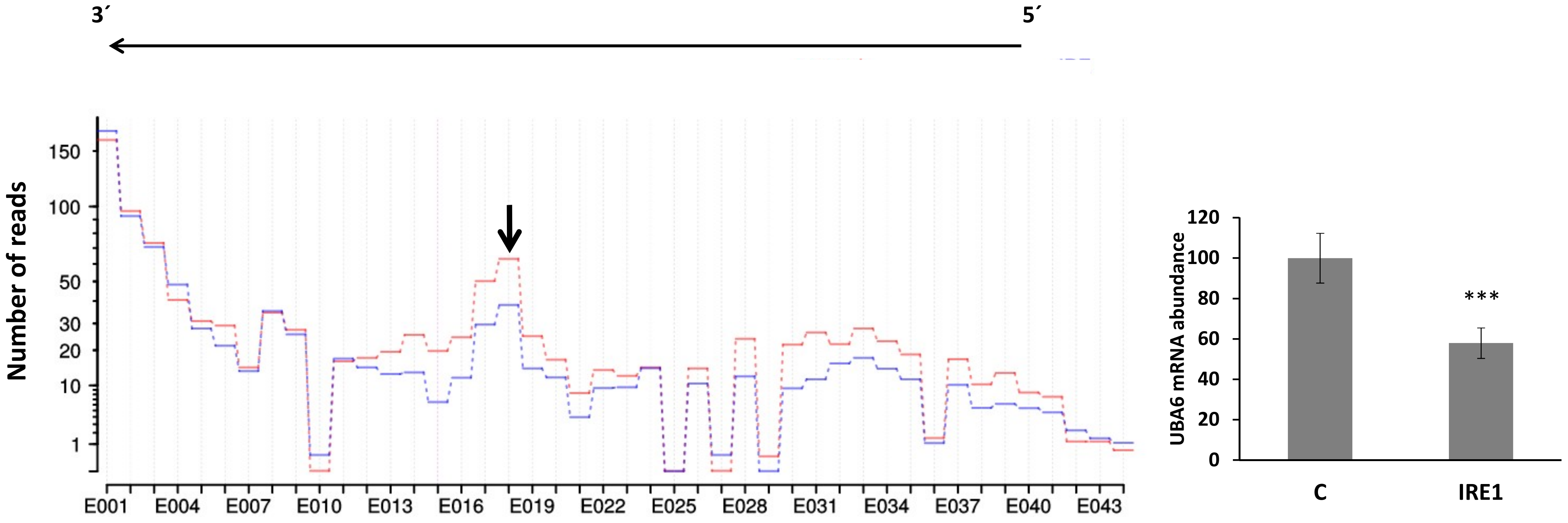
3´
5´
Number of reads
150
100
50
30
20
10
1
E001
E004
E007
E010
E013
E016
E019
E022
E025
E028
E031
E034
E037
E040
E043
UBA6 mRNA abundance
120
100
80
60
40
20
0
***
C
IRE1

IKZF1

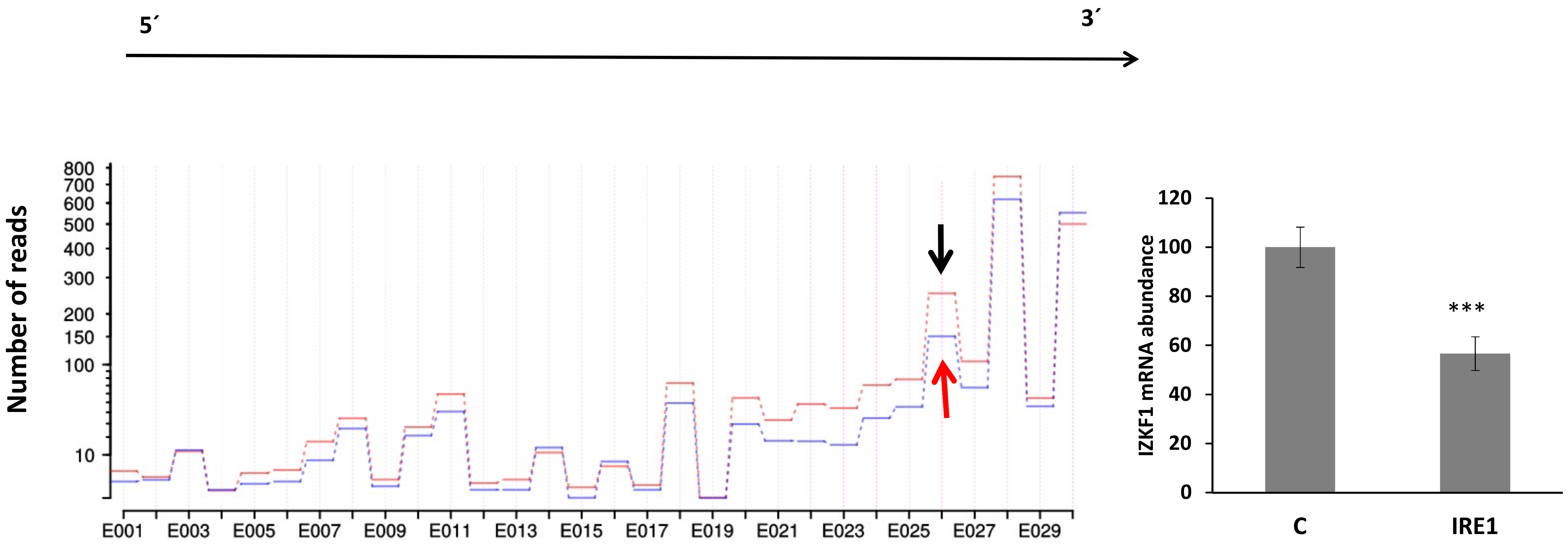
5´
3´
Number of reads
800
700
600
500
400
300
200
150
100
10
E001
E003
E005
E007
E009
E011
E013
E015
E017
E019
E021
E023
E025
E027
E029
IZKF1 mRNA abundance
120
100
80
60
40
20
0
***
C
IRE1

PSMD1

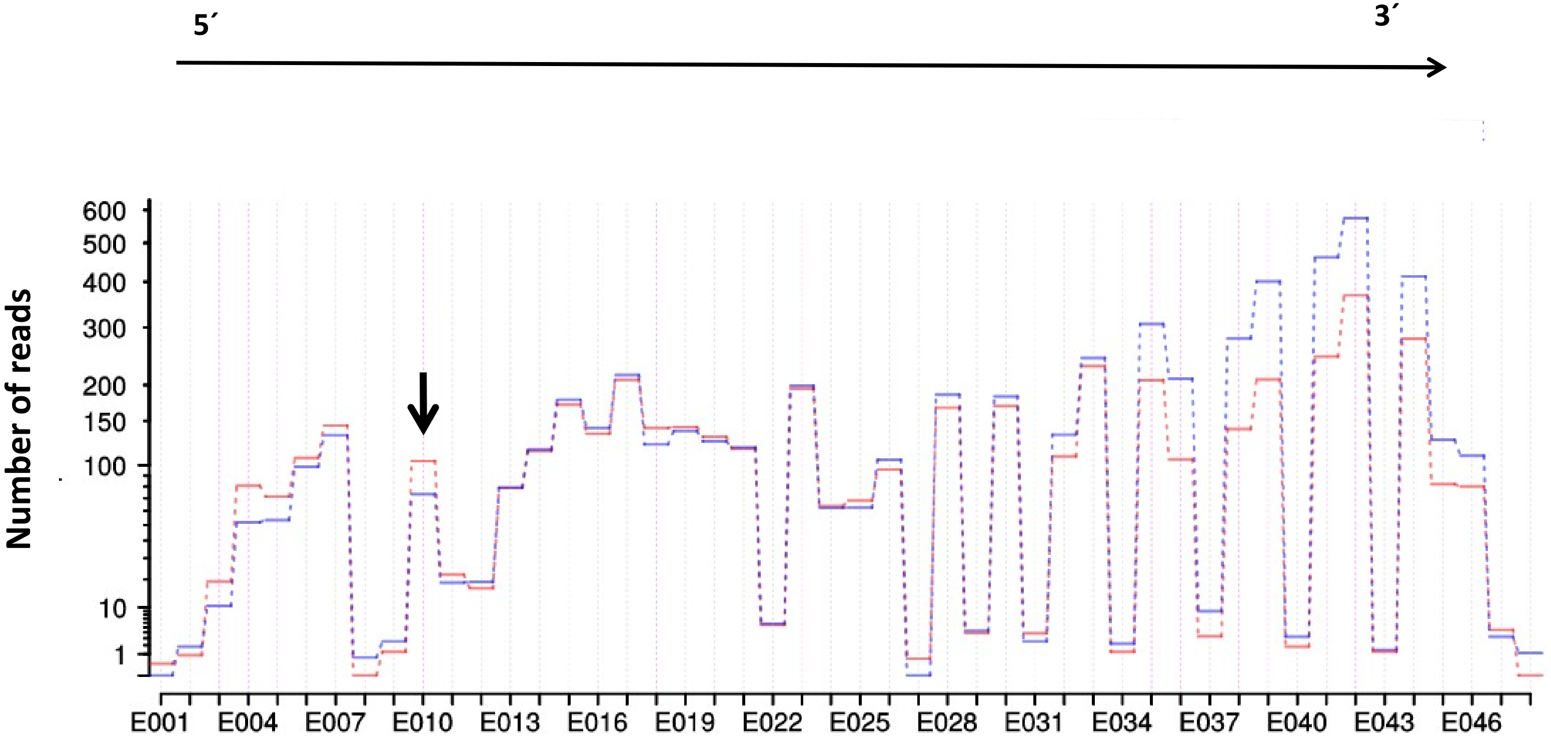
5´
3´
Number of reads
600
500
400
300
200
150
100
10
1
E001
E004
E007
E010
E013
E016
E019
E022
E025
E028
E031
E034
E037
E040
E043
E046

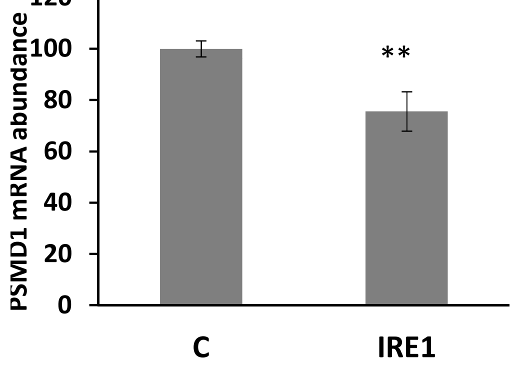
PSMD1 mRNA abundance
120
100
80
60
40
20
0
**
C
IRE1

CUL5

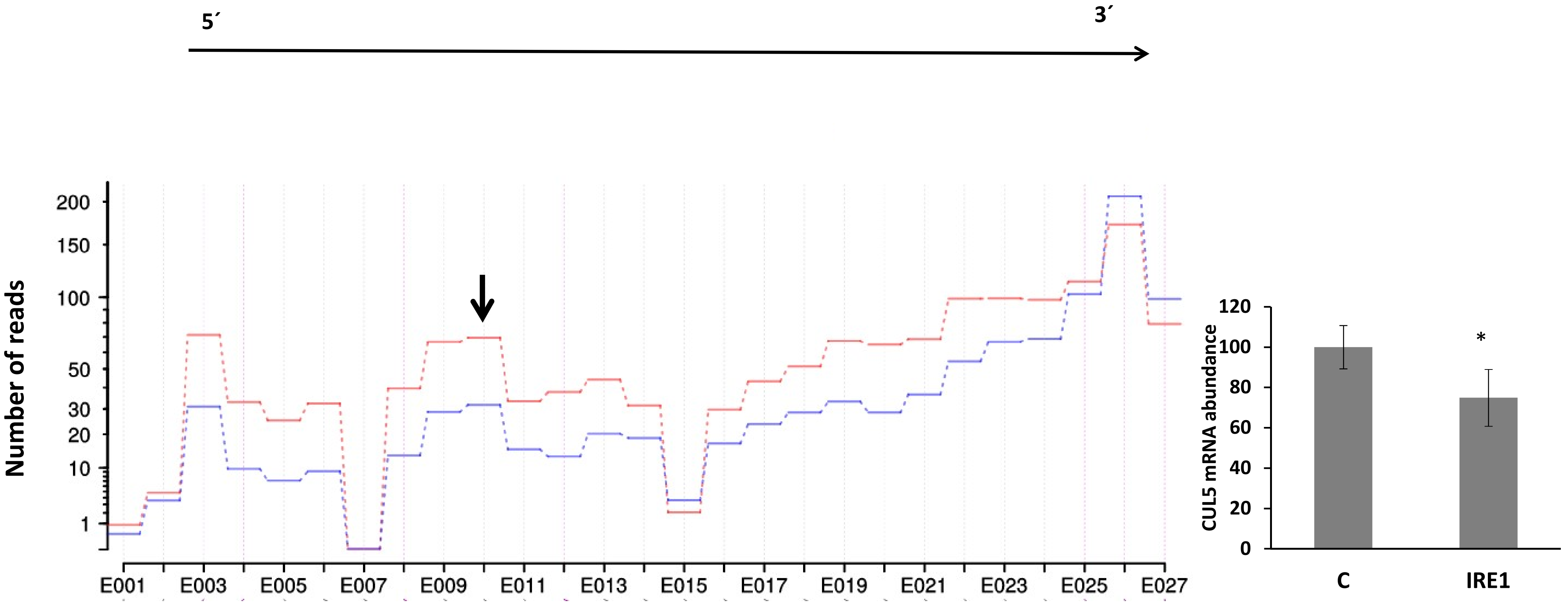
5´
3´
Number of reads
200
150
100
50
30
20
10
1
E001
E003
E005
E007
E009
E011
E013
E015
E017
E019
E021
E023
E025
E027
CUL5 mRNA abundance
120
100
80
60
40
20
0
*
C
IRE1

ERAP1

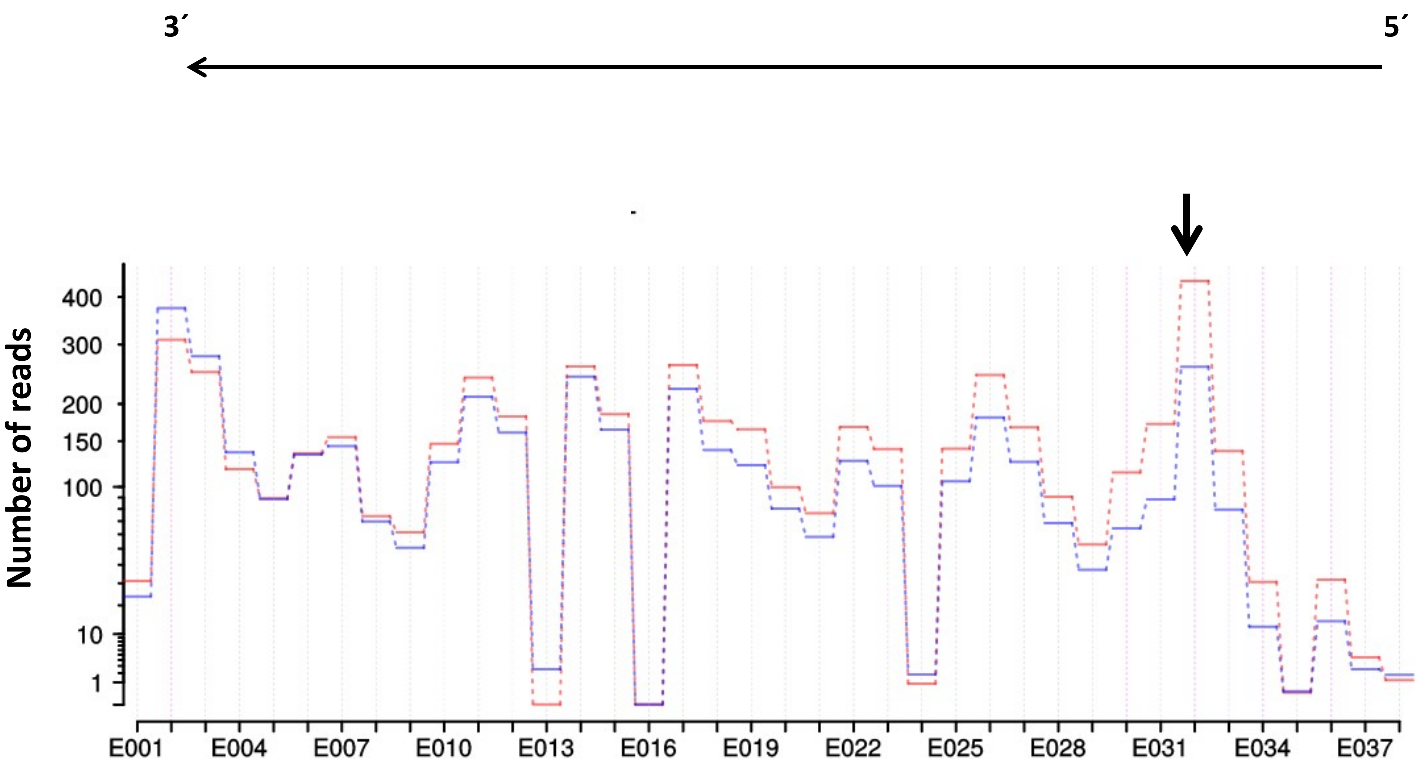
3´
5´
Number of reads
400
300
200
150
100
10
1
E001
E004
E007
E010
E013
E016
E019
E022
E025
E028
E031
E034
E037

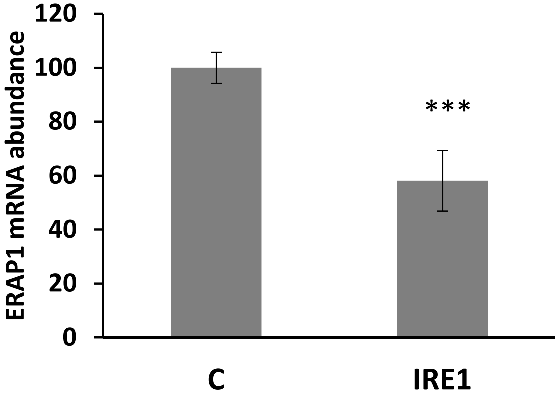
ERAP1 mRNA abundance
120
100
80
60
40
20
0
***
C
IRE1

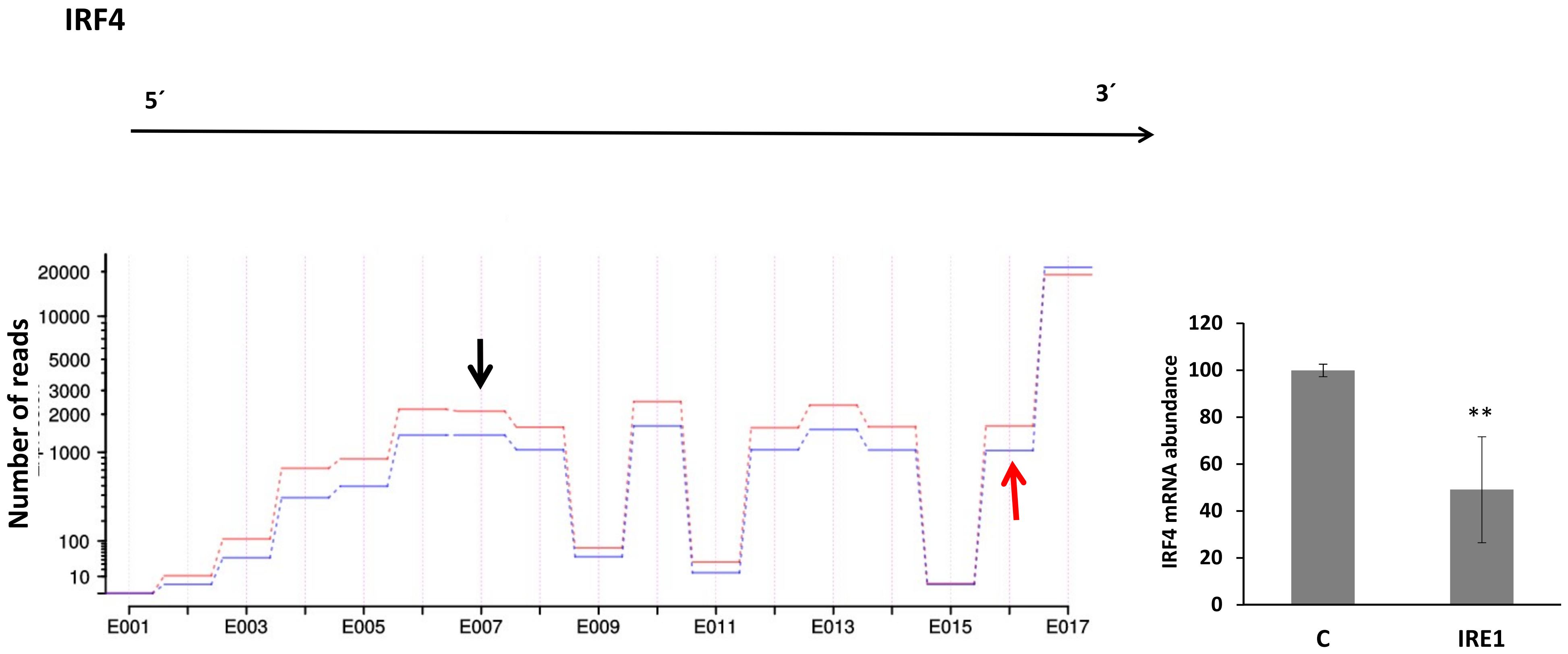
IRF4
5´
3´
Number of reads
20000
10000
5000
3000
2000
1000
100
10
E001
E003
E005
E007
E009
E011
E013
E015
E017
IRF4 mRNA abundance
120
100
80
60
40
20
0
**
C
IRE1
